# Effectiveness and cost-effectiveness of six GLP-1RAs for treatment of Chinese type 2 diabetes mellitus patients that inadequately controlled on metformin: a micro-simulation model

**DOI:** 10.3389/fpubh.2023.1201818

**Published:** 2023-09-06

**Authors:** Shuai Yuan, Yingyu Wu

**Affiliations:** Department of Pharmacoeconomics, School of International Pharmaceutical Business, China Pharmaceutical University, Nanjing, Jiangsu, China

**Keywords:** discrete event micro-simulation model, effectiveness, cost-effectiveness, type 2 diabetes mellitus, glucagon-like peptide-1 receptor agonists

## Abstract

**Objective:**

To systematically estimate and compare the effectiveness and cost-effectiveness of the glucagon-like peptide-1 receptor agonists (GLP-1RAs) approved in China and to quantify the relationship between the burden of diabetic comorbidities and glycosylated hemoglobin (HbA1c) or body mass index (BMI).

**Methods:**

To estimate the costs (US dollars, USD) and quality-adjusted life years (QALY) for six GLP-1RAs (exenatide, loxenatide, lixisenatide, dulaglutide, semaglutide, and liraglutide) combined with metformin in the treatment of patients with type 2 diabetes mellitus (T2DM) which is inadequately controlled on metformin from the Chinese healthcare system perspective, a discrete event microsimulation cost-effectiveness model based on the Chinese Hong Kong Integrated Modeling and Evaluation (CHIME) simulation model was developed. A cohort of 30,000 Chinese patients was established, and one-way sensitivity analysis and probabilistic sensitivity analysis (PSA) with 50,000 iterations were conducted considering parameter uncertainty. Scenario analysis was conducted considering the impacts of research time limits. A network meta-analysis was conducted to compare the effects of six GLP-1RAs on HbA1c, BMI, systolic blood pressure, and diastolic blood pressure. The incremental net monetary benefit (INMB) between therapies was used to evaluate the cost-effectiveness. China’s *per capita* GDP in 2021 was used as the willingness-to-pay threshold. A generalized linear model was used to quantify the relationship between the burden of diabetic comorbidities and HbA1c or BMI.

**Results:**

During a lifetime, the cost for a patient ranged from USD 42,092 with loxenatide to USD 47,026 with liraglutide, while the QALY gained ranged from 12.50 with dulaglutide to 12.65 with loxenatide. Compared to exenatide, the INMB of each drug from highest to lowest were: loxenatide (USD 1,124), dulaglutide (USD −1,418), lixisenatide (USD −1,713), semaglutide (USD −4,298), and liraglutide (USD −4,672). Loxenatide was better than the other GLP-1RAs in the base-case analysis. Sensitivity and scenario analysis results were consistent with the base-case analysis. Overall, the price of GLP-1RAs most affected the results. Medications with effective control of HbA1c or BMI were associated with a significantly smaller disease burden (*p* < 0.05).

**Conclusion:**

Loxenatide combined with metformin was identified as the most economical choice, while the long-term health benefits of patients taking the six GLP-1RAs are approximate.

## 1. Introduction

The epidemic of type 2 diabetes mellitus (T2DM) in China is the largest in the world and continues to increase. In 2018, the prevalence of T2DM in Chinese adults reached approximately 12.4%, with only 32.9% receiving antidiabetic treatment, among whom the rate of achieving glycaemic control was approximately 50.1% (1). The high incidence and incurability of diabetes impose an economic burden on patients and the healthcare system. By 2020, diabetes-related health expenditure reached USD 109.0 billion, ranking second to the United States (2). Therefore, how to maximize the health of diabetic patients in the allocation of limited health resources is a common concern of the country, society, and patients.

Metformin is considered the first-line treatment for diabetes, which helps reduce hepatic glucose production and insulin resistance (3). However, when metformin monotherapy or multiple oral glucose-lowering drugs cannot help T2DM patients achieve the aim of glycemic control, it is necessary to start using second-line drugs (4). Glucagon-like peptide-1 receptor agonists (GLP-1RAs) combined with metformin is the most common second-line regimen currently (5). The hypoglycemic mechanism of GLP-1RAs is to inhibit glucagon secretion from pancreatic ɑ cells, thereby inhibiting abnormal hepatic glycogen output. They are effective antihyperglycemic treatments that can reduce the risk of hypoglycemia and can also promote weight loss (6). Since the first GLP-1RAs were successfully approved by the U.S. Food and Drug Administration (FDA) in 2005, a growing number of countries have adopted GLP-1RAs combined with metformin as the mainstay treatment for patients with T2DM which is inadequately controlled on metformin (7). In China, the currently marketed GLP-1RAs include exenatide, benaglutide, lixisenatide, loxenatide, dulaglutide, liraglutide, and semaglutide, which all are recommended to be used as treatments for patients with T2DM which is inadequately controlled on metformin by 2022 Chinese clinical guidelines (8).

After the new round of national basic medical insurance access negotiations in 2021, all of the above-referred GLP-1RAs have entered the drug list of the national medical insurance in China, the price of these drugs changed significantly (for example, the price reduction of dulaglutide injection reached 64.5%; the price reduction of liraglutide was 52.7%; the price reduction of lixisenatide reached 43%; and the price reduction of semaglutide injection reached 60%) and patients have more options (8). However, to our knowledge, no published clinical or economic studies have directly compared the cost-effectiveness of these treatments in China. Therefore, we aimed to estimate and compare the effectiveness and cost-effectiveness of the GLP-1RAs approved in China for T2DM patients with ineffective glycemic control with metformin, so as to provide reliable evidence for policymakers in adjusting the drug prices and for clinicians and patients in medication.

## 2. Materials and methods

### 2.1. Model overview

**Figure 1:**
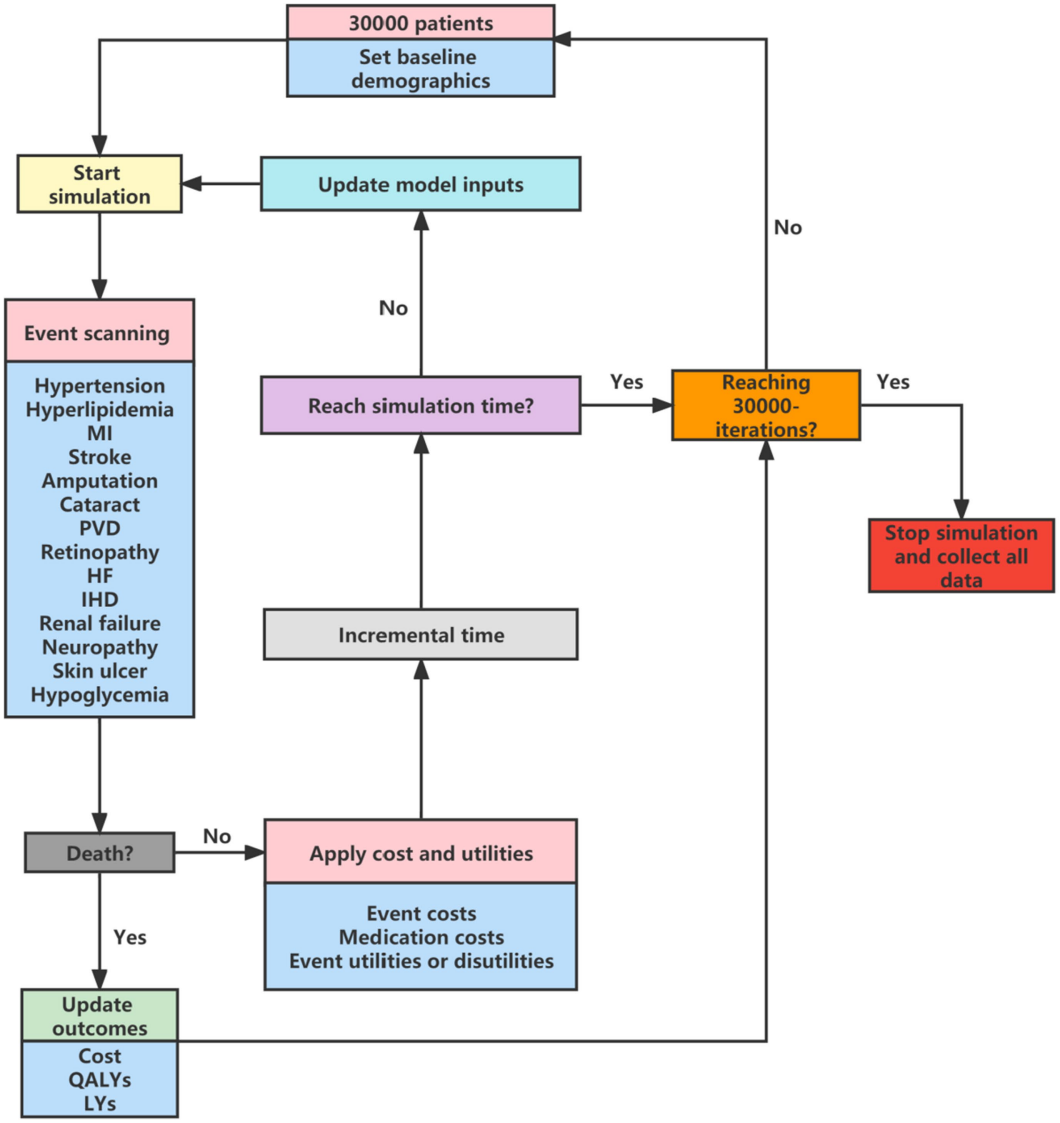
Model structure for CHIME-CE. MI, myocardial infarction; PVD, peripheral vascular disease; HF, heart failure; IHD, ischemic heart disease; QALY, quality-adjusted life years; LYs, life-years; CHIME, Chinese Hong Kong Integrated Modeling and Evaluation simulation model; CHIME-CE, CHIME-based discrete event micro-simulation cost-effectiveness model.

Our study was conducted based on a validated Chinese diabetes-related health outcomes model called the Chinese Hong Kong Integrated Modeling and Evaluation (CHIME) simulation model (9). The CHIME model is the first validated tool for predicting outcomes in Chinese patients with prediabetes and type 2 diabetes that was developed using Chinese data. The CHIME simulation model comprises 13 risk equations to predict all-cause mortality, diabetes-related macrovascular events (myocardial infarction, ischemic heart disease, heart failure, and cerebrovascular disease), microvascular events (peripheral vascular disease, neuropathy, amputation, ulcer of the skin, renal failure, cataracts, and retinopathy), and development of diabetes status. More details about the development of the CHIME model have been reported elsewhere (9). A CHIME-based discrete event micro-simulation cost-effectiveness model (CHIME-CE) was built to simulate the costs and outcomes for different treatments and long-term mortality was adjusted for diabetes all-cause mortality risk using population natural mortality (10). Natural mortality was extracted from China’s 6th National Census (11), the model structure is shown in Figure 1. A cohort of 30,000 patients was simulated and the cycle length was 1 year. This cost-effectiveness analysis was conducted from the perspective of the Chinese healthcare system, and a lifetime horizon was applied, with 99% of patients dying. All costs and health outputs were discounted at a rate of 5%. Our economic evaluation adhered to the Consolidated Health Economic Evaluation Reporting Standards 2022 (CHEERS 2022).

### 2.2. Target population and treatment strategies

The target population was in line with the population analyzed in the trials, that is, Chinese adults, aged between 50 and 60, with T2DM which was previously inadequately controlled on metformin and received GLP-1RAs plus metformin as second-line therapy. The patient characteristic profiles were assumed to be the means reported in the original RCTs. When data pertaining to a specific parameter that was used for estimating the complications, such as the usage of antihypertensives or insulin, were not available, information from the Chinese Clinical Management System was used as a references (9, 12). More details are presented in Tables 1, 2.

**Table 1:** Parameters.

Item	Mean (range)	Distribution	Sources
Clinical-related parameters			
Age, years (Mean/SD)	55 (44–66)	gamma	(13–22)
Proportion of female	0.44 (0.35–0.52)	beta	(13–22)
Duration of diabetes (years)	6 (4.3–7.6)	gamma	(13–22)
Proportion of current smoker	0.14 (0.11–0.16)	beta	(9)
Proportion of past smoker	0.19 (0.15–0.23)	beta	(9)
Baseline HbA1c (%, before intervention)	8.5 (7.6–9.4)	gamma	(22)
Baseline BMI (kg/m^2^, before intervention)	26.5 (25–29.15)	gamma	(14)
Baseline SBP (mmHg, before intervention)	125.5 (112.95–138.05)	gamma	(14)
Baseline DBP (mmHg, before intervention)	78.5 (70.65–86.35)	gamma	(14)
Baseline HDL cholesterol (mmol/L, before intervention)	1.3 (1.04–1.56)	gamma	(9)
Baseline LDL cholesterol (mmol/L, before intervention)	3 (2.4–3.6)	gamma	(9)
Baseline Triglycerides (mmol/L, before intervention)	1.6 (1.28–1.92)	gamma	(9)
Baseline Hemoglobin (g/L, before intervention)	13.7 (10.96–16.44)	gamma	(9)
Baseline White cell count (×10^9^, before intervention)	8 (6.4–9.6)	gamma	(9)
Baseline eGFR (mL/min/1.73m^2^, before intervention)	92.2 (73.76–110.64)	gamma	(9)
Insulin usage ratio	0.00 (0.00–0.00)	beta	assumed
Non-insulin hypoglycemic agent’s usage ratio	1 (1–1)	beta	assumed
Antihypertensives usage ratio	0.35 (0.28–0.42)	beta	(9)
Statins usage ratio	0.08 (0.06–0.09)	beta	(9)
Proportion of atrial fibrillation	0.02 (0.01–0.02)	beta	(9)
Proportion of MI	0.02 (0.01–0.02)	beta	(9)
Proportion of ischemic heart disease	0.03 (0.02–0.03)	beta	(9)
Proportion of heart failure	0.02 (0.01–0.02)	beta	(9)
Proportion of cerebrovascular disease	0.04 (0.03–0.05)	beta	(9)
Proportion of peripheral vascular disease	0 (0–0)	beta	(9)
Proportion of neuropathy	0 (0–0)	beta	(9)
Proportion of amputation	0 (0–0)	beta	(9)
Annual incidence of severe hypoglycemia	0.01 (0.01–0.01)	beta	(23)
Proportion of mild CKD	0.24 (0.19–0.29)	beta	(24)
Proportion of mild moderate CKD	0.03 (0.02–0.03)	beta	(24)
Proportion of moderate severe CKD	0 (0–0)	beta	(24)
Proportion of severe CKD	0 (0–0)	beta	(24)
Proportion of renal failure	0.01 (0.01–0.01)	beta	(9)
Proportion of retinopathy	0.01 (0.01–0.01)	beta	(9)
Proportion of cataract	0.04 (0.03–0.05)	beta	(9)
Proportion of ulcer of skin	0 (0–0)	beta	(9)
AE rates for exenatide			
Nausea	0.38 (0.3–0.45)	beta	(13)
Diarrhea	0.14 (0.11–0.17)	beta	(13)
Upper respiratory tract infection	0.03 (0.02–0.03)	beta	(13)
Vomiting	0.13 (0.1–0.16)	beta	(13)
Dizziness	0.01 (0.01–0.01)	beta	(13)
Sinusitis	0.02 (0.01–0.02)	beta	(13)
Hypoglycemia	0.07 (0.06–0.09)	beta	(13)
Back pain	0.02 (0.01–0.02)	beta	(13)
AE rates for loxenatide			
Nausea	0.01 (0–0.01)	beta	(14)
Diarrhea	0.03 (0.03–0.04)	beta	(14)
Vomiting	0.01 (0–0.01)	beta	(14)
Hypoglycemia	0.02 (0.02–0.03)	beta	(14)
AE rates for semaglutide			
Nausea	0.21 (0.17–0.25)	beta	(15)
Diarrhea	0.14 (0.11–0.17)	beta	(15)
Upper respiratory tract infection	0.03 (0.02–0.04)	beta	(15)
Vomiting	0.1 (0.08–0.12)	beta	(15)
Dizziness	0.07 (0.06–0.08)	beta	(15)
Sinusitis	0.05 (0.04–0.06)	beta	(15)
Constipation	0.05 (0.04–0.06)	beta	(15)
Hypoglycemia	0.02 (0.02–0.02)	beta	(15)
Lipase increased	0.07 (0.05–0.07)	beta	(15)
Decreased appetite	0.11 (0.07–0.11)	beta	(15)
AE rates for dulaglutide			
Nausea	0.19 (0.15–0.23)	beta	(18)
Diarrhea	0.14 (0.11–0.17)	beta	(18)
Vomiting	0.10 (0.08–0.12)	beta	(18)
Dizziness	0.07 (0.05–0.08)	beta	(18)
Sinusitis	0.08 (0.06–0.09)	beta	(18)
Decreased appetite	0.08 (0.07–0.10)	beta	(18)
AE rates for liraglutide			
Nausea	0.14 (0.11–0.16)	beta	(16)
Diarrhea	0.09 (0.07–0.11)	beta	(16)
Vomiting	0.06 (0.05–0.07)	beta	(16)
Dizziness	0.06 (0.05–0.07)	beta	(16)
Sinusitis	0.05 (0.04–0.06)	beta	(16)
Constipation	0.05 (0.04–0.06)	beta	(16)
Decreased appetite	0.05 (0.04–0.06)	beta	(16)
AE rates for lixisenatide			
Nausea	0.26 (0.21–0.32)	beta	(19–21)
Diarrhea	0.11 (0.09–0.13)	beta	(19–21)
Vomiting	0.11 (0.09–0.13)	beta	(19–21)
Hypoglycemia	0.02 (0.02–0.03)	beta	(19–21)
Cost-related parameter			
Discount	0.05 (0.00–0.08)	beta	(25)
Cost_exenatide_600μg	60.96 (48.77–60.96)	gamma	(26)
Cost_liraglutide_18mg	50.65 (40.52–50.65)	gamma	(26)
Cost_loxenatide_100μg	16.44 (13.15–16.44)	gamma	(26)
Cost_dulaglutide_1.5 mg	22.26 (17.81–22.26)	gamma	(26)
Cost_semaglutide_2mg	71.54 (57.23–71.54)	gamma	(26)
Cost_lixisenatide_20ug	40.04 (32.03–40.04)	gamma	(26)
Cost_Metformin_500mg	0.77 (0.51–1.07)	gamma	(26)
Cost_Insulin glargine_300IU	10.76 (10.01–12.1)	gamma	(26)
Dose_metformin/mg per day	1,365 (933.8–1796.2)	gamma	(22)
Dose_Insulin glargine/IU per day	8 (4–16)	gamma	assumed
Cost_Antidiabetic therapy per year (5 years<disease duration <10 years)	438.3 (109.58–913.13)	gamma	(27, 28)
Cost_Antidiabetic therapy per year (disease duration ≥10 years)	657.45 (255.68–1168.8)	gamma	(27, 28)
Cost_MI event year	7800.45 (6769.75–8831.25)	gamma	(28)
Cost_MI per following year	455.4 (288.6–622.2)	gamma	(28)
Cost_Stroke event year	3339.86 (2593.34–5497.3)	gamma	(28)
Cost_Stroke per following year	506.9 (445.9–828)	gamma	(28)
Cost_CHF first year	5254.89 (4203.91–6305.87)	gamma	(29)
Cost_CHF per following year	2787.69 (2230.15–3345.23)	gamma	(29)
Cost_Renal failure per year	13803.2 (13153.81–14569.21)	gamma	(28)
Cost_Skin ulcer event year	2612.19 (2200.91–3023.47)	gamma	(30)
Cost_Care for Skin per following year	793.31 (356.22–1230.4)	gamma	(30)
Cost_PVD event year	3193.64 (2554.91–3832.36)	gamma	(29)
Cost_PVD per following year	501.72 (401.37–602.06)	gamma	(29)
Cost_Amputation event year	2376.36 (1901.09–2851.64)	gamma	(29)
Cost_Amputation per following year	2134.02 (1707.22–2560.82)	gamma	(29)
Cost_Neuropathy event year	2553.56 (2042.85–3064.28)	gamma	(29)
Cost_Neuropathy per following year	979.68 (783.74–1175.62)	gamma	(29)
Cost_Hypoglycemia per event	534.4 (400.8–667.9)	gamma	(31)
Cost_IHD event year	1966.04 (1816.09–2115.99)	gamma	(30)
Cost_IHD per following year	445.66 (322.42–568.9)	gamma	(30)
Cost_Retinopathy per event	939.34 (751.47–1127.21)	gamma	(32)
Cost_Cataract event year	1544.75 (1235.8–1853.7)	gamma	(29)
Cost_Cataract per following year	54.09 (43.27–64.9)	gamma	(29)
Cost_End-of-life	22987.5 (18,390–27,585)	gamma	(33)
Utility-related parameter			
Disutilities for each comorbidity			
Disu_MI hospitalization year	0.24 (0.19–0.29)	beta	(28)
Disu_MI after discharge	0.17 (0.14–0.2)	beta	(28)
Disu_Stroke hospitalization	0.19 (0.15–0.23)	beta	(28)
Disu_Stroke after discharge	0.11 (0.09–0.14)	beta	(28)
Disu_T2DM without complications	0.06 (0–0.264)	beta	(28)
Disu_CHF	0.25 (0.026–0.446)	beta	(28)
Disu_Renal failure	0.16 (0.09–0.14)	beta	(28)
Disu_Neuropathy	0.02 (0.007–0.037)	beta	(34)
Disu_Hypoglycemia	0.06 (0.042–0.071)	beta	(35)
Disu_Amputation	0.28 (0.22–0.34)	beta	(29)
Disu_Post Amputation	0.28 (0.22–0.34)	beta	(29)
Disu_Skin ulcer	0.06 (0.05–0.07)	beta	(30)
Disu_Retinopathy	0.02 (0.011–0.034)	beta	(34)
Disu_Cataract	0.02 (0.001–0.031)	beta	(34)
Disu_PVD	0.02 (0–0.125)	beta	(34)
Disu_IHD	0.02 (0–0.041)	beta	(34)
Disu_BMI_PER INCRE	0.05 (0.04–0.06)	beta	(30)
Utility_BMI_PER DECRE	0.02 (0.01–0.02)	beta	(30)
Grade 1-2 AEs	0.014 (0.008–0.02)	beta	(36)

AEs, adverse events; BMI, body mass index; CKD, chronic kidney disease; MI, myocardial infarction; PVD, peripheral vascular disease; CHF, heart failure; IHD, ischemic heart disease; Disu, disutility; SD, standard deviation; INCRE, increase; DECRE, decrease; HbA1c, glycosylated hemoglobin; SBP, systolic pressure; DBP, diastolic pressure; HDL, high density lipoprotein; LDL, low density lipoprotein; eGFR, estimated glomerular filtration rate.

**Table 2:** Information for RCT.

RCT	Group	*N*	Sex (Percent of Male)	Region	Age/years	Weight/kg	Body-Mass Index/kg/m^2^	HbA1c/%	Fasting Plasma Glucose/mmol/L	Duration of Diabetes/years
SUSTAIN-7	semaglutide 1 mg	300	54	Global	58 ± 10	95.5 ± 20.9	34 ± 7	8.2 ± 0.9	9.8 ± 2.6	7.3 ± 5.7
	dulaglutide 1.5 mg	299	57	Global	57 ± 9	93.4 ± 21.8	33 ± 7	8.2 ± 0.9	9.6 ± 2.3	7.6 ± 5.6
AWARD-6	dulaglutide 1.5 mg	299	46	Global	57 ± 10	93.8 ± 18.2	34 ± 5	8.1 ± 0.8	9.3 ± 2.2	7.1 ± 5.4
	liraglutide 1.8 mg	300	50	Global	57 ± 10	94.4 ± 19.0	34 ± 5	8.1 ± 0.8	9.2 ± 2.3	7.3 ± 5.4
Exendin-4	Placebo BID intend-to-treat	113	67	Global	54 ± 9	100 ± 19	34 ± 6	8.2 ± 1.0	9.4 ± 2.2	6.6 ± 6.1
	5ug exenatide BID intend-to-treat	110	57	Global	53 ± 11	100 ± 22	34 ± 6	8.3 ± 1.1	9.8 ± 2.4	6.2 ± 5.9
	10ug exenatide BID intend-to-treat	113	68	Global	52 ± 11	101 ± 20	34 ± 6	8.2 ± 1.0	9.3 ± 2.6	4.9 ± 4.7
LEAD-2	liraglutide 1.8 mg	100	59	Global	57 ± 9	NA	30.9 ± 4.6	8.4 ± 1.0	10.1 ± 2.3	8 ± 5
	glimepiride	100	57	Global	57 ± 9	NA	31.2 ± 4.6	8.4 ± 1.0	10.0 ± 2.6	8 ± 5
	Placebo	100	60	Global	56 ± 9	NA	31.6 ± 4.4	8.4 ± 1.1	10.0 ± 2.3	8 ± 6
AWARD-5	dulaglutide 1.5 mg	304	48	Global	54 ± 10	87 ± 17	31 ± 5	8.1 ± 1.1	NA	7 ± 6
	Placebo	177	51	Global	55 ± 9	87 ± 17	31 ± 4	8.1 ± 1.1	NA	7 ± 5
GetGoal-X	lixisenatide 20 mg QD	318	47.5	Global	57.3 ± 9.2	94.0 ± 19.6	33.7 ± 6.3	8.03 ± 0.8	9.7 ± 2.0	6.8 ± 5.5
	exenatide 10 mg BID	316	59.2	Global	57.6 ± 10.7	96.1 ± 22.5	33.5 ± 6.5	8.02 ± 0.8	9.7 ± 2.3	6.8 ± 4.9
	All	634	53.3	Global	57.4 ± 9.9	95.0 ± 21.13	33.6 ± 6.4	8.02 ± 0.8	9.7 ± 2.1	6.8 ± 5.2
GetGoal-M	lixisenatide morning injection	255	38.4	Global	54.5 ± 9.2	90.1 ± 21.0	33.2 ± 6.9	8.0 ± 0.9	9.4 ± 2.2	6.2 ± 5.3
	lixisenatide evening injection	255	44.7	Global	54.8 ± 10.4	89.0 ± 20.7	32.5 ± 5.8	8.1 ± 0.9	9.3 ± 2.3	6.2 ± 5.4
	Combined placebo	170	47.6	Global	55.0 ± 9.4	90.4 ± 21.1	33.1 ± 6.5	8.1 ± 0.9	9.5 ± 2.3	5.9 ± 4.7
GetGoal-F1	lixisenatide one-step	161	44	Global	55.4 ± 8.9	90.3 ± 19.0	33.0 ± 5.8	8.0 ± 0.9	9.6 ± 2.0	5.8 ± 3.9
	lixisenatide two-step	161	45	Global	54.6 ± 8.9	88.0 ± 16.8	32.1 ± 4.8	8.1 ± 0.9	9.5 ± 2.5	6.0 ± 4.6
	Placebo combined	160	45	Global	58.2 ± 9.8	87.9 ± 17.3	32.4 ± 5.5	8.0 ± 0.8	9.5 ± 2.0	6.2 ± 4.7
Gao-2020	loxenatide 100 μg	179	57	Global	53.6 ± 10.5	71.2 ± 12.8	26.0 ± 3.5	8.5 ± 0.9	NA	4.3 ± 3.5
	Placebo	179	54.7	Global	52.3 ± 10.7	73.8 ± 14.1	26.9 ± 3.9	8.6 ± 0.9	NA	4.7 ± 3.4

QD, on prescription; Bid, twice a day.

A total of six GLP-1RAs were considered in our study, namely, exenatide (5 μg/bid for the first 4 weeks and then 10 μg/bid), liraglutide (0.6 mg/day in the first week, 1.2 mg/day in the second week, and 1.8 mg/day in the third week and beyond), loxenatide (5 μg/week), dulaglutide (1.5 mg/week), semaglutide (increase from a starting dose of 0.25 mg weekly to 1 mg, doubling the dose every 4 weeks), and lixisenatide (10 μg/day in the first week; 15 μg/day for the second week; and 20 μg/day in the third week and beyond). We did not consider benaglutide due to there being no relevant Phase 3 RCTs. All the patients were switched to insulin glargine U100 after 5 years, as their T2DM progressed with deterioration of their beta-cell function (37).

### 2.3. Clinical evidence

To inform the clinical inputs for the model, a systematic literature review was conducted in July 2021 to identify randomized controlled trials of relevant GLP-1RAs in the treatment of T2DM patients (38). A total of nine phase III RCTs with 3,850 patients were identified in the systematic literature review: Exendin-4 (13), Gao-2020 (14), SUSTAIN-7 (15), LEAD-2 (16), AWARD-5 (17), AWARD-6 (18), GetGoal-X (19), GetGoal-M (20), and GetGoal-F1 (21). The baseline characteristics of the patients in these trials (such as age, physical status, glycosylated hemoglobin (HbA1c), duration of diabetes, and fasting plasma glucose) were similar and therefore comparable. Additionally, a considerable proportion of Asian populations have been included in the global trials, therefore, they can serve as the source of clinical data for this study population. More details are provided in Table 2.

Network meta-analyses (NMAs) were then performed to assess the comparative efficacy of these six GLP-1RAs combined with metformin in the second-line treatment of T2DM. Four indicators were included in the analysis, namely the changing rates of glycated hemoglobin, body mass index (BMI), systolic blood pressure (SBP), and diastolic blood pressure (DBP) during the first 6 months. Considering that none of the included trials addressed the effect of lixisenatide on blood pressure, we used data from GetGoal-M-Asia (22) instead, as GetGoal-M and GetGoal-M-Asia were similar in design and had consistent results (e.g., 0.4% change in glycated hemoglobin with loxenatide compared to placebo in both studies during the first 6 months). The rates of change of the four indicators are presented in Supplementary Tables S1–S4. Random-effect Bayesian models estimated the risk ratios (RRs) of the changing rates between the six treatments via Markov chain Monte Carlo algorithms. The reference for glycated hemoglobin and BMI was set as lixisenatide in GetGoal-X, which was the trial with the largest sample size in our network, and semaglutide in SUSTAIN-7 was used as the reference for SBP and DBP for the same reason. Noninformative priors were used to allow the observed trial data to explain the effect estimates (39). We used the gemtc package (40) in R, version 4.1.0 (41) with four parallel Markov chains consisting of 50,000 samples after a 10,000 sample burn-in. The convergence of the Markov chains was checked by trace plots and Gelman-Rubin diagnostic statistics. The design-by-treatment approach was used to check the consistency in the entire NMA and the global inconsistency of the study was assessed with the *I*^2^ (chi-square test) statistic. The local inconsistency was evaluated by node-splitting analysis, which was used to assess the inconsistency of the model by separating evidence on a particular comparison into direct and indirect evidence. The significance level was *α* = 0.05 for statistical tests.

### 2.4. Costs and utilities

Only the direct costs of implementing each treatment were included considering the Chinese healthcare system. All cost data were converted to US dollars using the exchange rate from 2022 (1 USD = 6.693 CNY). The prices of the GLP-1RAs were obtained from the access price of the national basic medical insurance drug catalog in 2021, and the prices of insulin and metformin were derived from the median price of national centralized drug procurement in 2021 (26). The cost of antidiabetic treatment and blood glucose test strips related to T2DM was collected from a large screening study based on the Chinese population (27). We considered only adverse events (AEs) with rates >5% reported in the RCTs, and related treatment costs and duration of diabetes were from published articles. Other potential health resource consumption, such as outpatient treatment costs, hospital expenses related to diabetes complications, and end-of-life costs, were extracted from published cost-effectiveness research based on Chinese patients (28–33, 35). More details about the cost-related parameters are listed in Table 1.

Health state utilities were collected from a report of 12,583 Chinese patients with T2DM, and a validated Chinese EQ-5D-5L instrument was used to investigate the utility for diabetes mellitus and cardio-cerebrovascular disease without complications (42). Other utilities that were not reported in that study, such as ESRD and minor and major amputations, were retrieved from published studies based on Chinese patients (29, 30, 34, 35). Disutilities of AEs were from a Chinese-based CEA (36). All the utility-related parameters are shown in Table 1.

The short-term costs and utilities were calculated based on the microvascular or macrovascular events and AEs reported in the RCTs, and the long-term costs and utilities were predicted by the CHIME-CE model.

### 2.5. Cost-effectiveness analysis

The primary outputs of the model included costs, life years, quality-adjusted life years (QALY), net monetary benefit (NMB), incremental cost-effectiveness ratio (ICER), and incremental net monetary benefit (INMB). Considering the fact that the effectiveness of various treatments in our model is very similar, ICER was not used as the indicator in our sensitivity analysis. Instead, INMB was adopted. China’s *per capita* GDP in 2021 was used as the willingness-to-pay threshold (USD 12,728) in this study as recommended by the 2020 China Pharmacoeconomics Guideline (25).

### 2.6. Sensitivity analysis

Sensitivity analyses were performed to address the uncertainties in parameter values. We performed the one-way sensitivity analysis to test the sensitivity of results to changes in treatment effects-, costs-, and utilities-related parameters. Tornado graphs were plotted to visualize the parameters that had meaningful association with the results, and the INMB was used as a measure of cost-effectiveness. An INMB over 0 means the treatment was more cost-effective. A Monte Carlo simulation was performed for 50,000 iterations and the probabilistic sensitivity analysis was conducted. The gamma distribution was selected for cost; the beta distribution for probability, proportion, and utilities; and the normal distribution for the RRs between the treatments. All the parameters were adjusted within the reported 95% confidence intervals (CI) or assuming reasonable ranges of the base-case values. Cost-effectiveness acceptability curves were used to analyze the cost-effectiveness of each regimen with various Willingness-to-pay (WTP) thresholds.

### 2.7. Scenario analysis

Considering the impacts of research time limits, longer simulation time frames, while closer to patients’ lifetime costs and outcomes, also introduced more uncertainty. Therefore, we conducted a scenario analysis, comparing the cost, utilities, and NMB of each treatment when the research time limit was 10, 20, 30, and 40 years.

### 2.8. Relationship between HbA1c, weight control, and burden of diabetic comorbidities

Combined with the results of base-case analysis, we calculated the cost and negative effect of diabetes complications caused by different treatments. In addition, we used a generalized linear model (GLM) (43) with HbA1c and BMI as independent variables, costs and disutilities as dependent variables, and all other disease characteristics of patients as covariates (such as age, gender, blood pressure, disease history). According to the values, BMI and HbA1c were both converted into binary variables according to the relevant standards of the World Health Organization (WHO). Namely, HbA1c greater than 7% was regarded as insufficient glycated hemoglobin control (44), and BMI greater than 25 kg/m^2^ was considered to be ineffective in weight control (45). Through the GLM, the impacts of effective blood glucose or weight control on disease burden were analyzed.

## 3. Results

### 3.1. Network meta-analysis

Network plots are provided in Supplementary Figure S1. The RRs between the 6 treatments for glycated hemoglobin, BMI, SBP, and DBP are provided in Table 1. The results of global inconsistency and local inconsistency suggested that there was no inconsistency in all networks for the four indicators, and no significant differences were found between direct and indirect comparisons. More details can be found in Supplementary Figure S2.

### 3.2. Model validation

The model was validated against the China Health and Retirement Longitudinal Study (CHARLS) cohort which was a nationally representative longitudinal cohort of Chinese residents aged 45 and older and other outcomes models such as the United Kingdom Prospective Diabetes Study Outcomes Model 2 (UKPDS-OM2) and the Risk Equations for Complications of Type 2 Diabetes (RECODe). More details have been reported elsewhere (9). We summarise the main process for model validation in eMethods 1, 2 in the Supplementary material.

### 3.3. Base-case analysis

The results of the base-case analysis are shown in Table 3. The mean QALYs (95% CI) for patients who received dulaglutide, lixisenatide, exenatide, semaglutide, liraglutide, and loxenatide combined with metformin were 12.50 (12.46–12.54), 12.52 (12.48–12.56), 12.56 (12.51–12.60), 12.56 (12.51–12.61), 12.58 (12.54–12.62), and 12.65 (12.61–12.69), respectively, ranked from least to most effective. The mean costs (95% CI) for patients who received loxenatide, exenatide, dulaglutide, lixisenatide, semaglutide, and liraglutide combined with metformin were USD 42,092 (41,765–42,377), 42,114 (41,796–42,444), 42,763 (42,479–43,066), 44,016 (43,081–43,737), 46,414 (46,103–46,734), and 47,026 (46,685–47,341), respectively, ranked from least to most costly. The mean INMB (95% CI) for patients who received loxenatide, exenatide, dulaglutide, lixisenatide, semaglutide, and liraglutide combined with metformin was USD 118,865 (118,268–119,417), 117,736 (117,159–118,330), 116,325 (115,685–116,908), 116,029 (115,455–116,641), 113,442 (112,857–114,029), and 113,069 (112,496–113,653), respectively, ranked from highest to lowest. Compared with the INMB of exenatide combined with metformin, the difference in loxenatide, dulaglutide, lixisenatide, semaglutide, liraglutide was USD (95% CI) 1,124 (326–1,988), −1,418 (−2,209–594), −1,713 (−2,569–−815), −4,298 (−5,121–−3,465), and −4,672 (−5,519–−3,835), respectively, ranked from highest to lowest. Loxenatide was identified as the most cost-effective choice. The ICERs of each treatment compared to exenatide are presented in Supplementary Table S8. A breakdown of diabetes complication-related costs and utilities for all treatments is presented in Table 4.

**Table 3:** Base-case analysis results.

Drug	Exenatide	Liraglutide	Loxenatide	Dulaglutide	Semaglutide	Lixisenatide
Cost (USD, 95% CI)	42,114 (41,796–42,444)	47,026 (46,685–47,341)	42,092 (41,765–42,377)	42,763 (42,479–43,066)	46,414 (46,103–46,734)	44,016 (43,081–43,737)
Life-years (year, 95% CI)	23.87 (23.75–23.99)	23.86 (23.74–23.98)	24.01 (23.89–24.13)	23.67 (23.56–23.80)	23.74 (23.62–23.85)	23.79 (23.67–23.91)
Utility (QALY, 95% CI)	12.56 (12.51–12.60)	12.58 (12.54–12.62)	12.65 (12.61–12.69)	12.50 (12.46–12.54)	12.56 (12.51–12.61)	12.52 (12.48–12.56)
NMB (USD, 95% CI)	117,736 (117,159–118,330)	113,069 (112,496–113,653)	118,865 (118,268–119,417)	116,325 (115,685–116,908)	113,442 (112,857–114,029)	116,029 (115,455–116,641)
INMB^#^ (USD, 95% CI)	--	-4,672 (−5,519–−3,835)	1,124 (326–1988)	−1,418 (−2,209–594)	−4,298 (−5,121–−3,465)	−1713 (−2,569–−815)

#exenatide was used as the reference. CI, confidence interval; QALY, quality-adjusted life years; NMB, net monetary benefit; INMB, increased net monetary benefit.

**Table 4:** Cost (USD) and disutility (QALY) breakdown results.

Parameters	Amputation	Cataract	MI	PVD	Retinopathy	HF	IHD	Renal failure	Stroke	Neuropathy	Ulcer	Hypoglycemia	Sum
Dulaglutide													
Cost breakdown	93.30	286.90	465.01	138.49	46.74	930.87	702.91	5890.51	750.11	140.23	228.73	67.422	9741.24
Disutility breakdown	0.012	0.027	0.079	0.003	0.001	0.078	0.021	0.067	0.110	0.003	0.014	0.000	0.42
Liraglutide													
Cost breakdown	96.96	289.22	467.37	139.20	50.39	1641.47	700.97	5919.25	724.05	125.95	245.51	67.296	10467.62
Disutility breakdown	0.013	0.027	0.080	0.003	0.001	0.078	0.021	0.067	0.106	0.003	0.016	0.000	0.41
Loxenatide													
Cost breakdown	84.71	278.21	445.44	150.14	43.25	907.02	715.82	6040.80	715.51	291.81	239.05	68.091	9979.85
Disutility breakdown	0.011	0.026	0.076	0.004	0.001	0.076	0.022	0.068	0.105	0.003	0.015	0.000	0.41
Semaglutide													
Disutility breakdown	79.59	271.93	451.15	136.21	41.86	888.99	687.61	5598.65	698.56	136.03	198.93	67.049	9256.56
Disutility breakdown	0.010	0.025	0.075	0.003	0.001	0.075	0.021	0.063	0.102	0.003	0.012	0.000	0.39
Exenatide													
Disutility breakdown	122.61	299.34	487.43	156.71	55.90	946.97	717.78	6608.31	746.22	145.34	252.77	67.671	10607.06
Disutility breakdown	0.016	0.028	0.083	0.004	0.001	0.079	0.022	0.075	0.110	0.003	0.016	0.000	0.44
Lixisenatide													
Cost breakdown	135.67	300.39	480.52	150.71	57.10	950.71	685.60	6314.71	754.29	153.94	275.20	67.346	10326.18
Disutility breakdown	0.018	0.028	0.081	0.004	0.001	0.080	0.021	0.071	0.111	0.003	0.017	0.000	0.44

Disu, disutility; MI, myocardial infarction; PVD, peripheral vascular disease; HF, heart failure; IHD, ischemic heart disease.

### 3.4. Sensitivity analysis

#### 3.4.1. One-way sensitivity analysis

**Figure 2:**
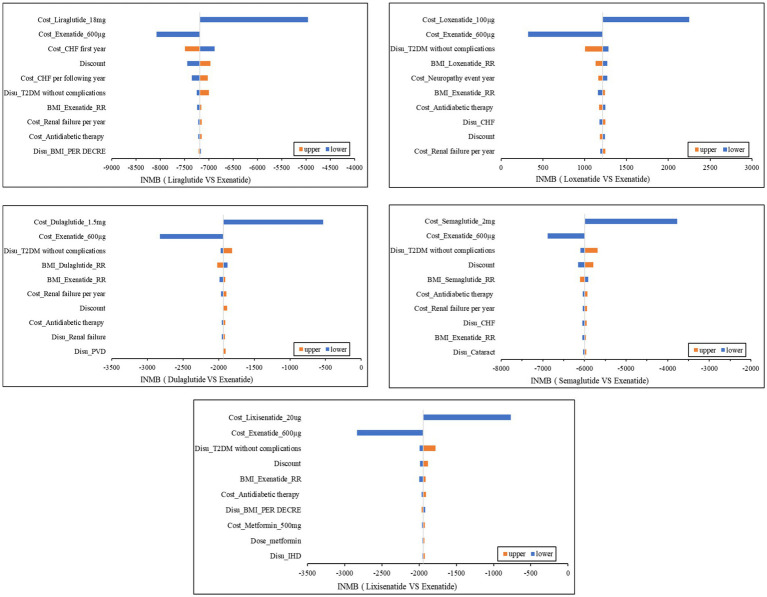
Tornado Graphs. MI, myocardial infarction; PVD, peripheral vascular disease; HF, heart failure; IHD, ischemic heart disease; T2DM, type 2 diabetes; Disu, disutility; RR, risk ratios; BMI, Body Mass Index; Disu, disutilities; T2DM, type 2 diabetes mellitus; INMB, increased net monetary benefit; CHF, congestive heart failure.

We used the INMB to measure economic effectiveness. The NMB of exenatide combined with metformin was used as the comparison, and tornado graphs of the other five treatments (Figure 2) compared with exenatide combined with metformin showed the 10 parameters that had the greatest impact on cost-effectiveness. On the whole, the costs of GLP-1RAs and discounts had the greatest impact on the INMBs. Although each parameter fluctuated, the INMB of exenatide combined with metformin was always >0 compared with the alternatives except loxenatide combined with metformin. Loxenatide was more economical compared with exenatide combined with metformin.

#### 3.4.2. Probabilistic sensitivity analysis

**Figure 3:**
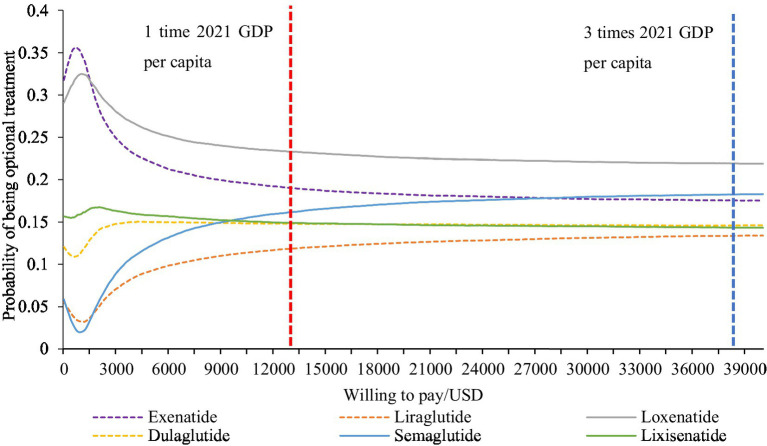
Cost-effectiveness acceptability curves (CEAC). USD, US dollars.

According to the cost-effectiveness acceptability curves (Figure 3), exenatide combined with metformin was the most economical option for Chinese T2DM patients when WTP was lower than USD 8800. When WTP was between USD 8800 and 40,000 (approximately 3 times GDP *per capita*), loxenatide combined with metformin was the most economical option. Under the chosen threshold (USD 12,728), loxenatide had an 18.92% probability of being the most economical treatment, and exenatide was suboptimal, with a probability of 18.67%. The results of Probabilistic Sensitivity Analysis (PSA) revealed that loxenatide was slightly more economical than exenatide. The cost-effectiveness of lixisenatide and dulaglutide combined with metformin were similar, with probabilities of 17.59 and 16.98%, respectively. Semaglutide combined with metformin ranked fifth, with a 14.15% probability, which was slightly better than the sixth-ranked liraglutide (13.67% probability of being the most cost-effective option).

#### 3.4.3. Scenario analysis

According to Supplementary Figure S3 and Table S6, when the time limit equaled 10, 20, 30, or 40 years, the costs, utilities, and NMB for all the treatments were consistent with the base-case results. Specifically, the costs of exenatide and loxenatide were the lowest among the six options over time. Loxenatide with higher prices, when compared to exenatide, was the cost-saving option in the long run due to better control effects on HbA1c. The utilities of these options were roughly the same. Exenatide was the most economical treatment in the first 10 years, while loxenatide was the more cost-effective drug after then owing to better control of glycated hemoglobin.

### 3.5. Relationship between HbA1c, weight control, and burden of diabetic comorbidities

According to Table 4, the diabetes complication-related costs (USD 9256) and disutilities (0.39) of semaglutide combined with metformin were the lowest and were combined with the best controlling effects for glycosylated hemoglobin, BMI, and blood pressure from among the six treatments, followed by dulaglutide (cost, USD 9741; disutilities, 0.42), loxenatide (cost, USD 9980; disutilities, 0.41), lixisenatide (cost, USD 10326; disutilities, 0.44), liraglutide (cost, USD 10467; disutilities, 0.41), exenatide (cost, USD 10607; disutilities, 0.44). In the lifetime limits, compared to effective glycated hemoglobin control (HbA1c < 7%), patients taking medications with poor glycated hemoglobin control would cost $667 more, together with 0.013 QALYs lost. Compared to effective weight control (BMI < 25 kg/m^2^), medications that are effective in weight control would save patients $706, together with 0.017 QALYs gained. More details are provided in Supplementary Table S7.

## 4. Discussion

Using the CHIME-CM model, the cost-effectiveness of six GLP-1RAs (exenatide, liraglutide, loxenatide, dulaglutide, semaglutide, and lixisenatide) combined with metformin as second-line therapy in patients with poor glycemic control on metformin alone was compared. Base-case analysis results showed that loxenatide combined with metformin appeared to be the most economical option. Compared with the INMB of exenatide combined with metformin, the difference in loxenatide, dulaglutide, lixisenatide, semaglutide, liraglutide was USD (95% CI) 1,124 (326–1,988), −1,418 (−2,209–594), −1,713 (−2,569–−815), −4,298 (−5,121–−3,465), and −4,672 (−5,519–−3,835), respectively, ranked from highest to lowest. The one-way sensitivity analysis and scenario analysis results were generally consistent with the base-case analysis results. Overall, the one-way sensitivity analysis results indicated that the prices of these six GLP-1RAs affected the conclusion the most. The results of the PSA suggested that loxenatide combined with metformin had the greatest probability of being the most economical treatment, exenatide was suboptimal. The scenario analysis results suggested that, although exenatide was the most economical treatment in the first 10 years, loxenatide was the most cost-effective drug in the long term.

Due to the relatively low burden of T2DM, although the impact of the included treatments on the four indicators varied, a similar loss of utilities was caused. As can be seen from Table 4, the disutilities of the six treatments were almost the same. Furthermore, death risk was another indicator that had an important impact on outcomes. However, we found that there were no significant differences in the survival time of patients under the six combination regimens. Therefore, the costs of different options would play a crucial role in CEA, drugs with lower prices and comorbidity treatment costs tended to be more cost-effective. According to Figure 2, we can find that the impact of GLP-1RAs prices was greater than the latter. All six drugs have entered the China basic medical insurance catalog after great price cuts, so their prices are not very different, which could be the reason why the cost-effectiveness of the six treatments in the PSA is relatively close. Another reason for the small differences in the cost-effectiveness of the six options in the PSA is that the uncertainties of transition probabilities were considered. Readers can pay more attention to the PSA results as each parameter in the base-case analysis had certain uncertainties, we carried out 50,000 iterations while allowing each parameter to fluctuate to reduce the uncertainty of the base-case analysis results.

As the WTP threshold increased, the cost-effectiveness of high-priced GLP-1RAs, such as semaglutide, continued to improve. However, considering the relatively low economic burden of diabetes, it is unreasonable to set the threshold too high. Based on this, this study set the threshold of WTP to the lower limit of the guideline-recommended threshold. To conclude, loxenatide with a cheaper price and better control of glycated hemoglobin was the optimal choice for patients with T2DM which was previously inadequately controlled by metformin.

Additionally, the diabetes complication-related costs and utilities of semaglutide combined with metformin were USD 9256 and 0.39 QALYs, which were both the lowest among the six treatments. Effective control of HbA1c or BMI was associated with significant cost-saving and utility-gained for diabetic comorbidities. T2DM patients should strictly control their glycosylated hemoglobin and body weight, and adopt an active and healthy lifestyle to reduce the occurrence of comorbidities. The national health system should formulate scientific chronic disease management policies, such as establishing electronic files to monitor and inform patients of glycosylated hemoglobin, blood pressure, weight, etc., in real time.

To the best of our knowledge, existing diabetes outcomes models mainly include the UKPDS-OM2 (46), RECODe model (47), the BRAVO risk engine (48), the IMS CORE Diabetes Model (49), and CDC-RTI (50). There are some differences between the UKPDS and CHIME-CE. The UKPDS model is based on a 1970s UK cohort, while the CHIME-CE model is based on the nationwide Chinese population. Additionally, the sample size of CHIME was almost 10 times larger than that of the UKPDS. This indicates that CHIME-CE may have better calibration, reliability, and validity compared to the UKPDS model. Also, studies have shown that the UKPDS overestimates the absolute risks of coronary heart disease and stroke among Chinese T2DM patients (51). The more recent RECODe model for 10 years risks was developed from a trial in the United States/Canada (52). CDC-RTI, CORE, and BRAVO were all developed from trials conducted in European or North American settings. Therefore, existing diabetes outcomes models may not be able to be applied to Chinese populations except the CHIME model. CHIME is the first specialized simulation model for predicting the progression of diabetes and related outcomes in Chinese patients developed using Hong Kong Hospital Authority Clinical Management System data, which is one of the largest Chinese electronic health informatics systems with detailed clinical records. A study showed that the CHIME model is a validated tool for predicting the progression of diabetes and its outcomes among Chinese patients compared to UKPDS-OM2 and RECODe (9).

Currently, there are few research articles on the cost-effectiveness of GLP-1RAs. Hu et al. (53) evaluated the cost-effectiveness of semaglutide, dulaglutide, and extended-release exenatide in treating patients with T2DM which cannot be controlled with metformin-based background therapy. They found that dulaglutide appeared to be the most cost-effective option, which was consistent with our findings. Zhang et al. (54) evaluated the cost-effectiveness of metformin combined with liraglutide or exenatide in Chinese patients with T2DM, based on the CORE Diabetes Model. However, due to the influence of basic medical insurance and centralized purchasing policies, the prices of drugs have changed dramatically since 2016. Therefore, their research results cannot be used as a reference. Gu et al. (2) estimated and compared the cost-effectiveness of 10 commonly used pharmacologic combination strategies including GLP-1RAs combined with metformin for T2DM but did not distinguish between GLP-1RAs.

### 4.1. Strengths and limitations

Compared with previous similar studies (2, 53, 54), our study has the following advantages. First, we are the first to systematically compare the efficacy and cost-effectiveness of GLP-1RAs approved in China, thereby providing comprehensive evidential support for clinical rational drug use and medical service decision-making. Second, we adopted the CHIME-CE model to predict outcomes which were developed based on a validated tool for predicting the progression of diabetes and its outcomes among Chinese patients. Compared with other models, CHIME-CE is the first model based on the Chinese population, which means that our model was more accurate in predicting the outcomes of Chinese T2DM patients. Third, compared with other studies discussing the cost-effectiveness of GLP-1RAs, we not only considered the effects of GLP-1RAs on HbA1c but also other indicators that affect the risk of comorbidities. Fourth, we considered the impact of short-term AEs on costs and utilities, which had been overlooked in previous studies (2, 28–30, 35, 37, 53, 54). Finally, we quantified the impacts of effective glycated hemoglobin control and weight control on disease burden in patients with diabetes.

This study also has the following limitations. There were no head-to-head studies that directly compared all the glucose-lowering treatments against each other which meant that we had to perform an NMA. Although the articles included in this study were of high quality, indirect comparisons still brought some uncertainty. More head-to-head RCTs are needed to validate the findings of our study. Due to the limitation of data, the cost of retinopathy per event was sourced from Taiwan. However, the sensitivity analysis results indicate that the uncertainty of this parameter did not affect the conclusions of this study. Then, we only considered partial transition probability-related parameters such as RRs to simplify model calculations in the one-way sensitivity analysis, which may have led to an excessive agreement with base-case analysis results. Furthermore, the sources of cost and utility parameters were not from the same patients, which may have led to bias in economic evaluation. Due to the limited information reported by the RCTs, in the subsequent years, HbA1c, BMI, SBP, and DBP were predicted by the CHIME model. However, our study population and the CHIME population somewhat differed, predicted results may be biased, and future studies should pay more attention to this issue. In addition, some clinical parameters were sourced from global RCTs, and although this was necessary, there may still be potential biases to a certain extent. We only considered AEs with an occurrence rate of 5% or higher, this may lead to a certain degree of underestimation of costs and overestimation of utility in the base-case analysis and scenario analysis results. Finally, under the current circumstances, conducting a subgroup-based cost-effectiveness analysis is challenging, and we did not study the availability and affordability of GLP-1RAs, implying that further research is required.

## 5. Conclusion

Loxenatide was identified as the most economical choice for patients with T2DM which is inadequately controlled on metformin, and exenatide was a close suboptimal regimen in China. The long-term health benefits of patients taking the six GLP-1RAs were approximated. The prices of GLP-1RAs are the main factors influencing cost-effectiveness. Diabetes complication-related costs and utilities of semaglutide were the lowest among the six treatments.

## Data availability statement

The original contributions presented in the study are included in the article/Supplementary material, further inquiries can be directed to the corresponding author.

## Author contributions

SY and YW had full access to all of the data in the study and take responsibility for the integrity of the data and the accuracy of the data analysis, concept and design, acquisition of data, statistical analysis, and supervision. SY: analysis and interpretation of data and drafting of manuscript. YW: critical revision of the manuscript for important intellectual content, obtaining funding, and administrative and technical support. All authors contributed to the article and approved the submitted version.

## Conflict of interest

The authors declare that the research was conducted in the absence of any commercial or financial relationships that could be construed as a potential conflict of interest.

## Publisher’s note

All claims expressed in this article are solely those of the authors and do not necessarily represent those of their affiliated organizations, or those of the publisher, the editors and the reviewers. Any product that may be evaluated in this article, or claim that may be made by its manufacturer, is not guaranteed or endorsed by the publisher.
