# Supplementary material for: Effectiveness and cost-effectiveness of six GLP-1RAs for treatment of Chinese type 2 diabetes mellitus patients that inadequately controlled on metformin: a micro-simulation model
Source: Front Public Health. 2023 Sep 6;11:1201818. doi: 10.3389/fpubh.2023.1201818 (PMC10513082; doi:10.3389/fpubh.2023.1201818)
Supplement: Supplementary file 1 [file Data_Sheet_1.docx]

**Supplementary Material**

[Table S1 HbA1c 1](#_Toc5627)

[Table S2 Body Mass Index 2](#_Toc19202)

[Table S3 Systolic Blood Pressure 2](#_Toc18949)

[Table S4 Diastolic Blood Pressure 3](#_Toc4452)

[Figure S1 Network plots 3](#_Toc20991)

[Figure S2 Results of Global Inconsistency and Local Inconsistency 4](#_Toc24632)

[Figure S3 Results of Scenario Analysis 10](#_Toc18654)

[Table S5 Relative Effect Estimates for All Possible Pairwise Treatment Comparisons (CI, confidence interval) 11](#_Toc2729)

[Table S6 Results of Scenario Analysis 13](#_Toc28935)

[Table S7 Relationship between HbA1c, Weight Control and Burden of Diabetic Comorbidities 13](#_Toc7647)

[Table S8 ICERs of Each Treatment in the Base-case Analysis 14](#_Toc3566)

[eMethod 1 Details for 13 Cumulative Risk Equations 15](#_Toc22186)

[eMethod 2 Model Validation 21](#_Toc31658)

### Table S1 HbA1c

| RCT | Group | Baseline HbA1c (%) | Rates of change during 0.5 years (post-intervention/pre-intervention) | |
| --- | --- | --- | --- | --- |
|  |  |  | Mean | SE |
| Exendin-4 | Treatment group | 8.2 | 90.49% | 12.96% |
|  | Control group | 8.2 | 100.98% | 12.96% |
| Loxenatide | Treatment group | 8.5 | 86.35% | 12.73% |
|  | Control group | 8.6 | 104.07% | 12.55% |
| SUSTAIN 7 | Treatment group | 8.2 | 78.39% | 10.77% |
|  | Control group | 8.2 | 82.84% | 11.60% |
| LEAD-2 | Treatment group | 8.4 | 88.10% | 11.90% |
|  | Control group | 8.4 | 101.19% | 11.90% |
| AWARD-5 | Treatment group | 8.1 | 84.94% | 10.76% |
|  | Control group | 8.1 | 100.37% | 11.50% |
| AWARD-6 | Treatment group | 8.1 | 82.47% | 10.67% |
|  | Control group | 8.1 | 83.21% | 10.69% |
| GetGoal-X | Treatment group | 8.0 | 90.16% | 11.10% |
|  | Control group | 8.0 | 88.03% | 11.08% |
| GetGoal-M | Treatment group | 8.0 | 88.75% | 13.97% |
|  | Control group | 8.1 | 95.06% | 12.88% |
| GetGoal-F1 | Treatment group | 8.1 | 90.12% | 15.66% |
|  | Control group | 8.0 | 95.00% | 15.81% |

SE, standard error;

### Table S2 Body Mass Index

| RCT | Group | Baseline BMI (kg/m^2^) | Rates of change during 0.5 years (post-intervention/pre-intervention) | |
| --- | --- | --- | --- | --- |
|  |  |  | Mean | SE |
| Exendin-4 | Treatment group | 34.0 | 97.23% | 5.26% |
|  | Control group | 34.0 | 99.70% | 3.19% |
| Loxenatide | Treatment group | 26.0 | 99.29% | 3.85% |
|  | Control group | 26.9 | 98.91% | 3.72% |
| SUSTAIN 7 | Treatment group | 33.6 | 93.51% | 4.22% |
|  | Control group | 33.1 | 96.58% | 4.30% |
| LEAD-2 | Treatment group | 30.9 | 96.82% | 2.27% |
|  | Control group | 31.6 | 98.35% | 3.30% |
| AWARD-5 | Treatment group | 31.0 | 96.21% | 3.79% |
|  | Control group | 31.0 | 98.19% | 3.97% |
| AWARD-6 | Treatment group | 33.5 | 96.91% | 4.06% |
|  | Control group | 33.6 | 96.18% | 4.04% |
| GetGoal-X | Treatment group | 33.7 | 96.85% | 4.36% |
|  | Control group | 33.5 | 95.86% | 4.25% |
| GetGoal-M | Treatment group | 33.2 | 97.78% | 4.08% |
|  | Control group | 33.1 | 98.23% | 3.89% |
| GetGoal-F1 | Treatment group | 32.1 | 96.93% | 5.77% |
|  | Control group | 32.4 | 98.18% | 5.76% |

SE, standard error; BMI, Body Mass Index.

### Table S3 Systolic Blood Pressure

| RCT | Group | Baseline SBP (mmHg) | Rates of change during 0.5 years (post-intervention/pre-intervention) | |
| --- | --- | --- | --- | --- |
|  |  |  | mean | SE |
| Loxenatide | Treatment group | 124.3 | 99.84% | 8%^#^ |
|  | Control group | 126.8 | 99.05% | 10%^#^ |
| SUSTAIN 7 | Treatment group | 133.0 | 96.32% | 10.03% |
|  | Control group | 132.0 | 97.80% | 9.82% |
| AWARD-5 | Treatment group | 129.0 | 98.68% | 9.46% |
|  | Control group | 128.0 | 99.14% | 9.35% |
| AWARD-6 | Treatment group | 132.2 | 97.46% | 9.16% |
|  | Control group | 130.9 | 97.85% | 9.26% |
| GetGoal-M-ASIA | Treatment group | 124.3 | 98.95% | 8%^#^ |
|  | Control group | 126.8 | 97.87% | 8%^#^ |
| LEAD-2 | Treatment group | 131.0 | 98.24% | 8%^#^ |
|  | Control group | 135.0 | 98.67% | 8%^#^ |
| GetGoal-X | Treatment group | 133.0 | 97.82% | 8%^#^ |
|  | Control group | 132.0 | 98.11% | 8%^#^ |

# Assumption, not reported in RCTs; SBP, systolic blood pressure.

### Table S4 Diastolic Blood Pressure

| RCT | Group | Baseline DBP (mmHg) | Rates of change during 0.5 years (post-intervention/pre-intervention) | |
| --- | --- | --- | --- | --- |
|  |  |  | mean | SE |
| Loxenatide | Treatment group | 77.1 | 99.22% | 8%^#^ |
|  | Control group | 79.3 | 98.61% | 8%^#^ |
| SUSTAIN 7 | Treatment group | 82.0 | 97.56% | 10.35% |
|  | Control group | 80.0 | 99.88% | 10.16% |
| AWARD-5 | Treatment group | 78.0 | 99.49% | 8.94% |
|  | Control group | 78.0 | 99.10% | 10.23% |
| AWARD-6 | Treatment group | 79.9 | 99.72% | 8.66% |
|  | Control group | 79.1 | 99.61% | 8.76% |
| GetGoal-M-ASIA | Treatment group | 77.1 | 97.80% | 8%^#^ |
|  | Control group | 79.3 | 97.98% | 8%^#^ |
| GetGoal-X | Treatment group | 82.0 | 97.80% | 8%^#^ |
|  | Control group | 80.0 | 98.38% | 8%^#^ |

# Assumption, not reported in RCTs; DBP, diastolic blood pressure.

### Figure S1 Network plots

1. **HbA1c**

**
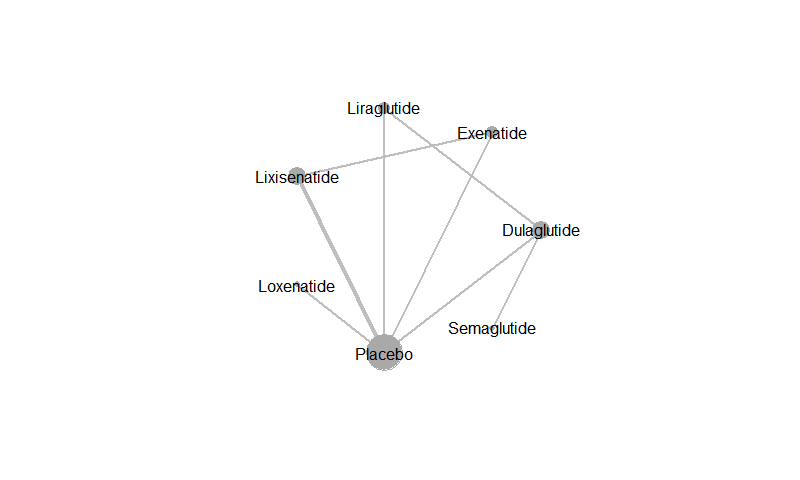
**

1. **Body Mass Index**

**
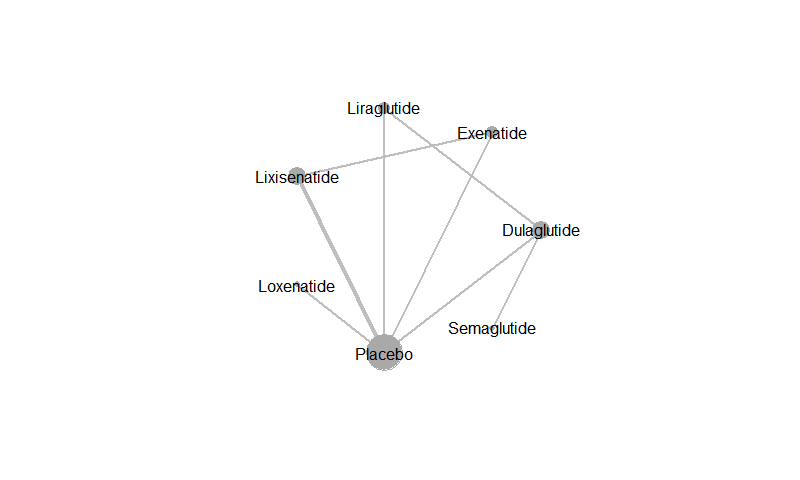
**

1. **Systolic Blood Pressure**

**
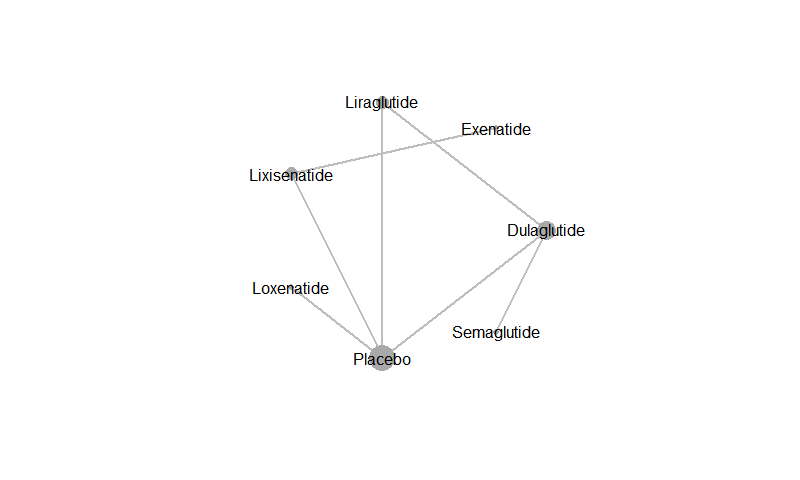
**

1. **Diastolic Blood Pressure**

**
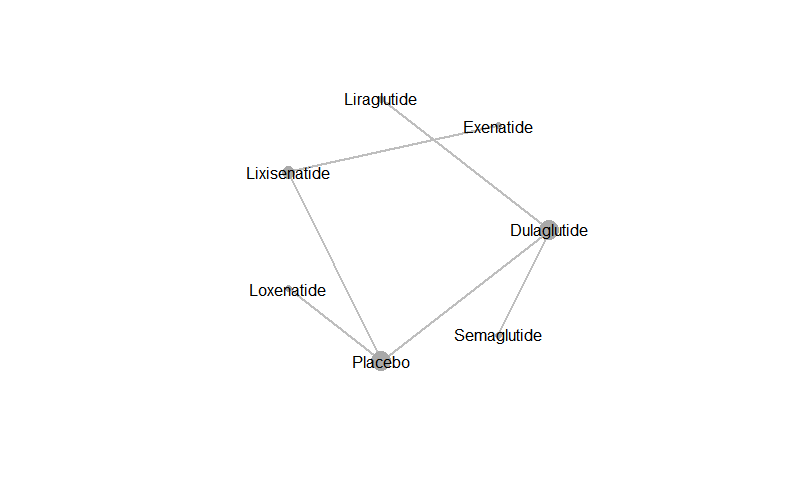
**

### Figure S2 Results of Global Inconsistency and Local Inconsistency

**A. HbA1c**

**
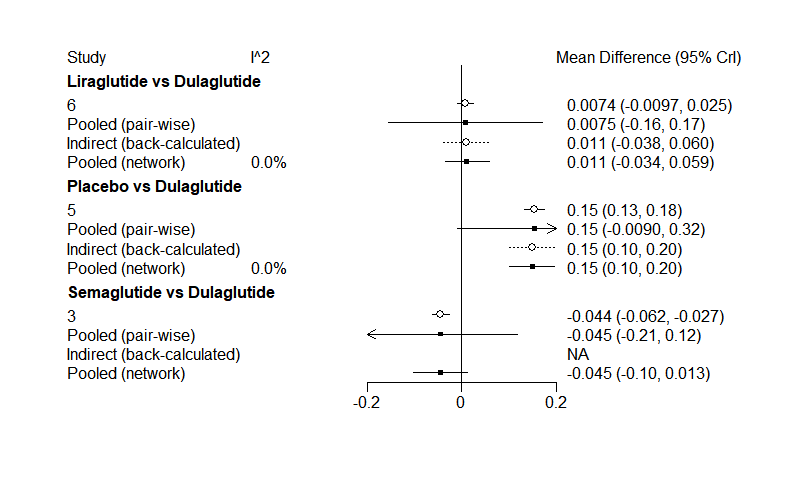
**

**
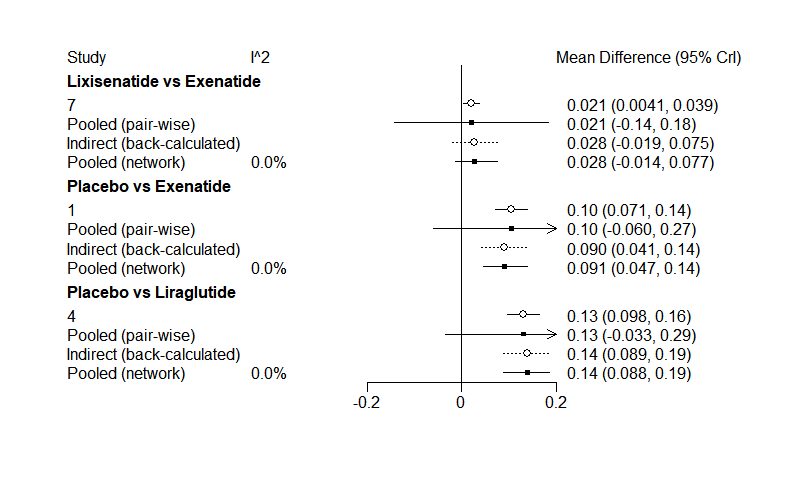
**

**
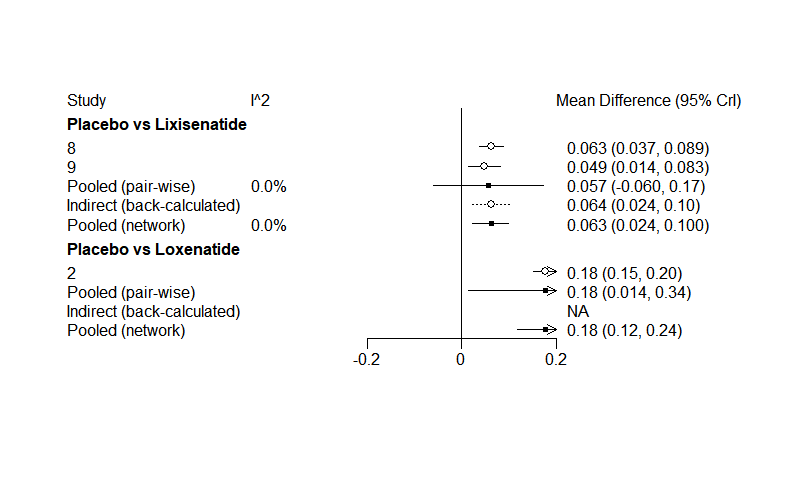
**

**
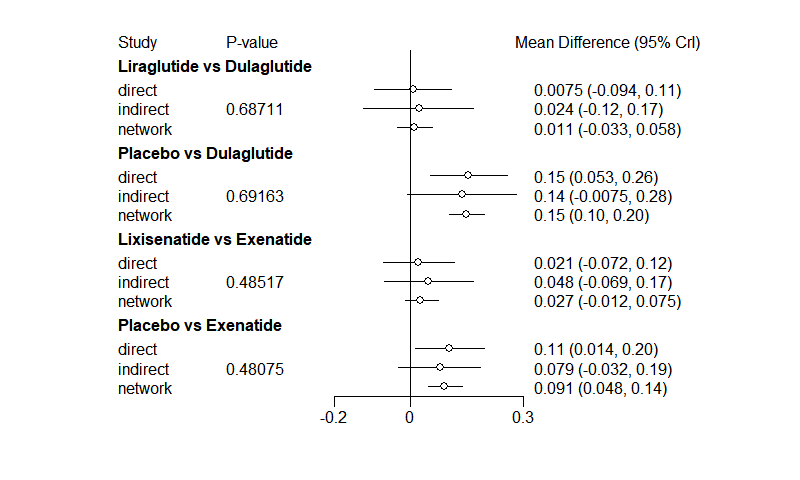
**

**
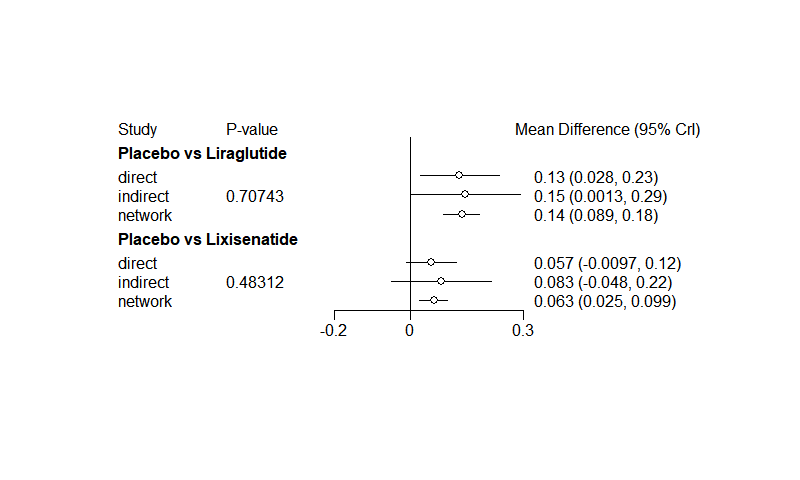
**

**B. Body Mass Index**

**
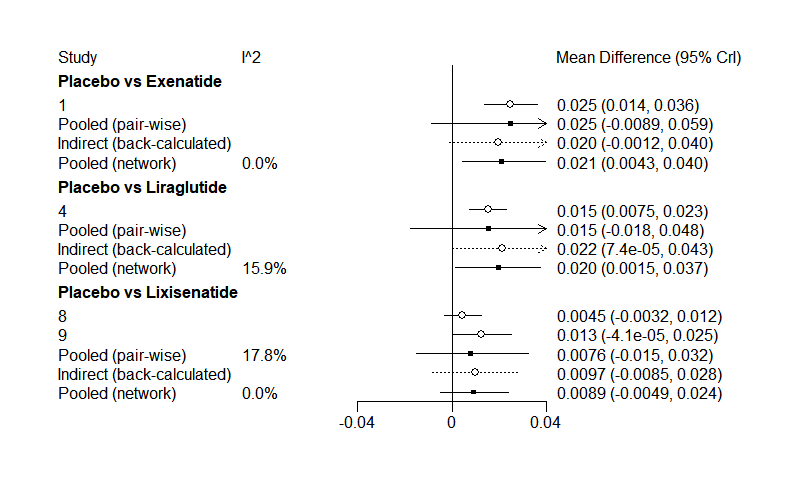
**

**
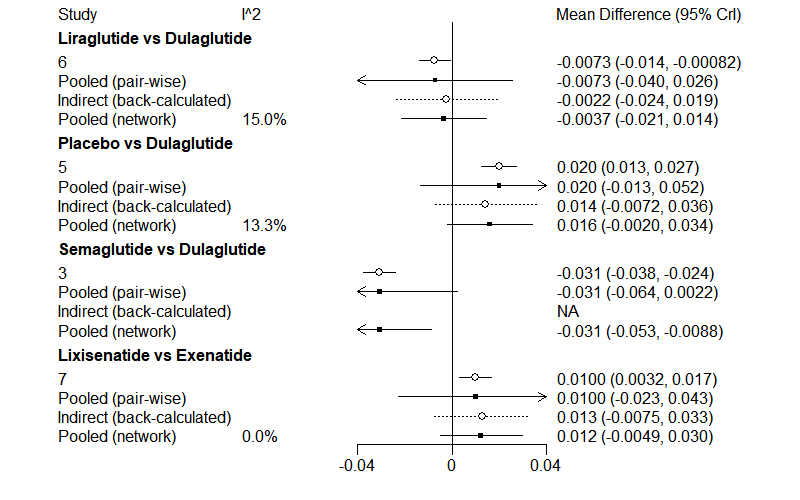
**

**
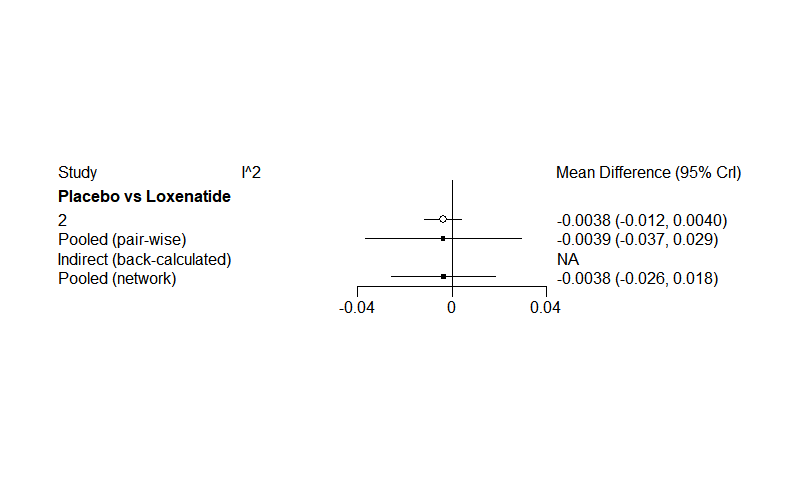
**

**
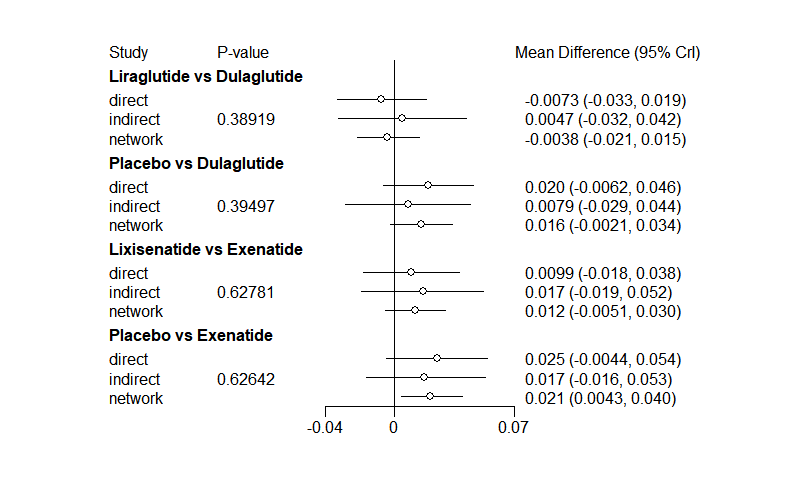
**

**
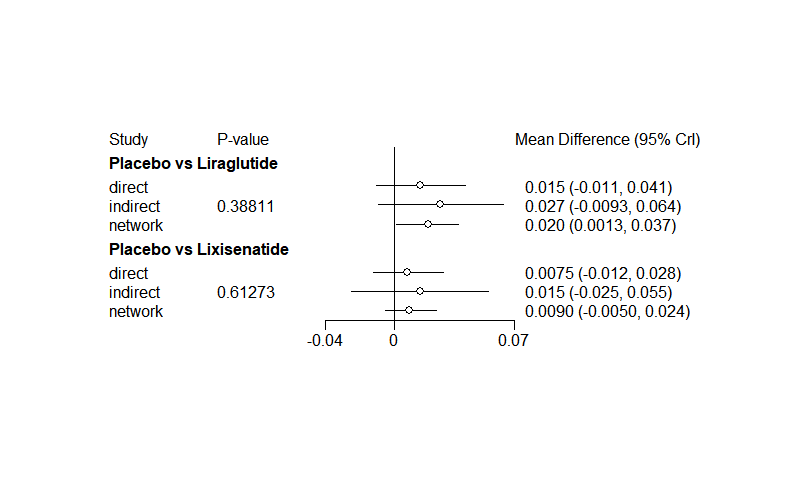
**

**C. Systolic Blood Pressure**

**
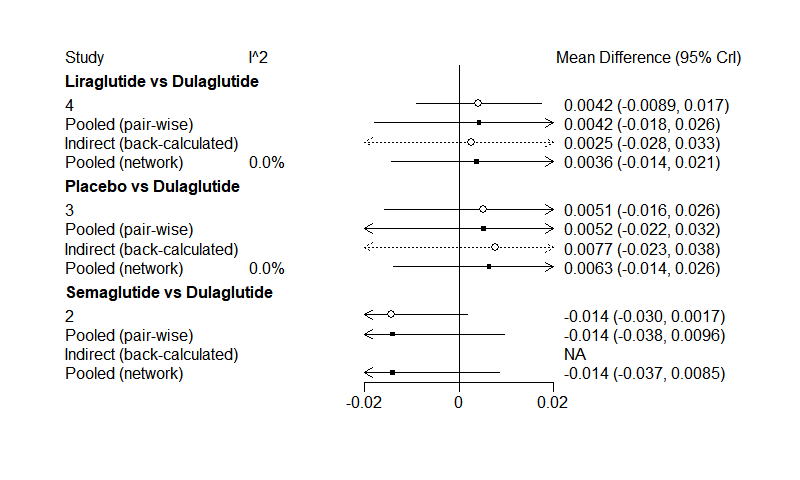
**

**
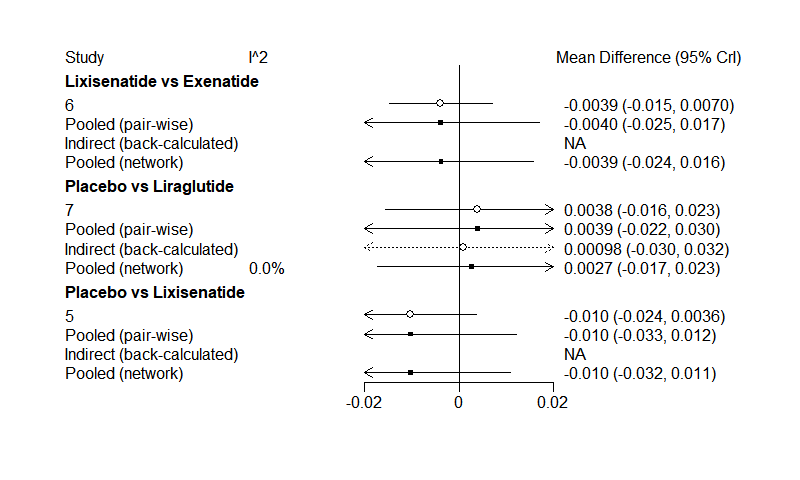
**

**
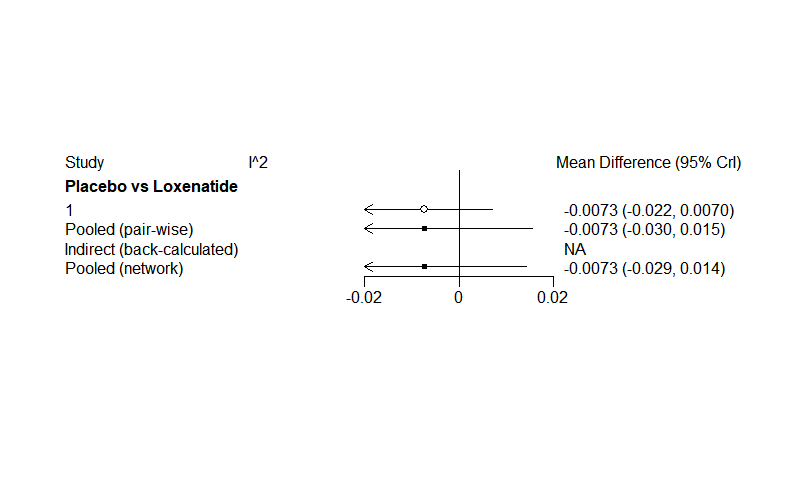
**

**
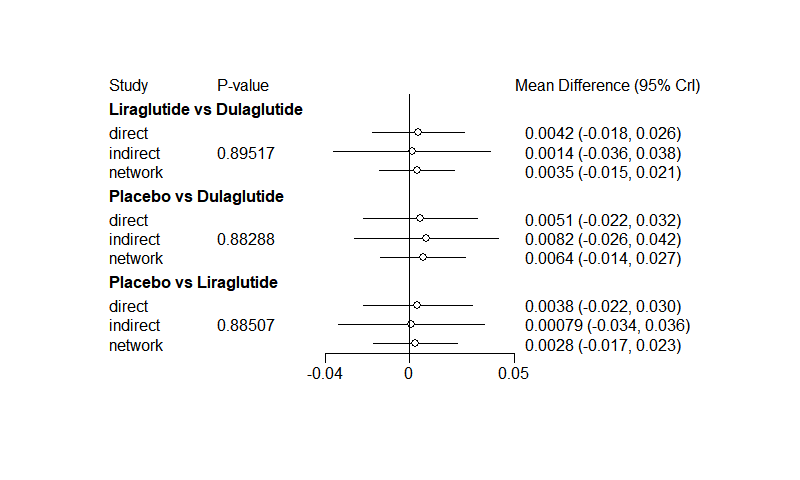
**

1. **Diastolic Blood Pressure: No source of heterogeneity**

### Figure S3 Results of Scenario Analysis

|  |
| --- |
|  |
|  |

NMB, net monetary benefit; USD, US dollars.

### Table S5 Relative Effect Estimates for All Possible Pairwise Treatment Comparisons (CI, confidence interval)

1. **Risk ratios for HbA1c (mean, 95% CI)**

| Dulaglutide |  |  |  |  |  |  |
| --- | --- | --- | --- | --- | --- | --- |
| 0.94 (0.88, 1.01) | Exenatide |  |  |  |  |  |
| 0.99 (0.94, 1.03) | 1.05 (0.98, 1.12) | Liraglutide |  |  |  |  |
| 0.92 (0.86, 0.97) | 0.97 (0.93, 1.01) | 0.93 (0.87, 0.99) | Lixisenatide |  |  |  |
| 1.03 (0.96, 1.11) | 1.09 (1.01, 1.17) | 1.04 (0.97, 1.12) | 1.12 (1.05, 1.21) | Loxenatide |  |  |
| 0.86 (0.82, 0.9) | 0.91 (0.87, 0.95) | 0.87 (0.83, 0.91) | 0.94 (0.91, 0.98) | 0.84 (0.79, 0.89) | Placebo |  |
| 1.05 (0.99, 1.11) | 1.11 (1.01, 1.21) | 1.06 (0.98, 1.14) | 1.14 (1.05, 1.24) | 1.02 (0.93, 1.11) | 1.21 (1.13, 1.31) | Semaglutide |

1. **Risk ratios for Body Mass Index (mean, 95% CI)**

| Dulaglutide |  |  |  |  |  |  |
| --- | --- | --- | --- | --- | --- | --- |
| 1.01 (0.98, 1.03) | Exenatide |  |  |  |  |  |
| 1 (0.99, 1.02) | 1 (0.97, 1.02) | Liraglutide |  |  |  |  |
| 0.99 (0.97, 1.02) | 0.99 (0.97, 1) | 0.99 (0.97, 1.01) | Lixisenatide |  |  |  |
| 0.98 (0.95, 1.01) | 0.98 (0.95, 1) | 0.98 (0.95, 1.01) | 0.99 (0.96, 1.01) | Loxenatide |  |  |
| 0.98 (0.97, 1) | 0.98 (0.96, 1) | 0.98 (0.96, 1) | 0.99 (0.98, 1) | 1 (0.98, 1.03) | Placebo |  |
| 1.03 (1.01, 1.05) | 1.03 (0.99, 1.06) | 1.03 (1, 1.06) | 1.04 (1, 1.07) | 1.05 (1.02, 1.09) | 1.05 (1.02, 1.08) | Semaglutide |

1. **Risk ratios for Systolic Blood Pressure (mean, 95% CI)**

| Dulaglutide |  |  |  |  |  |  |
| --- | --- | --- | --- | --- | --- | --- |
| 0.98 (0.95, 1.02) | Exenatide |  |  |  |  |  |
| 1 (0.98, 1.01) | 1.02 (0.98, 1.05) | Liraglutide |  |  |  |  |
| 0.98 (0.96, 1.01) | 1 (0.98, 1.02) | 0.99 (0.96, 1.02) | Lixisenatide |  |  |  |
| 0.99 (0.96, 1.02) | 1.01 (0.97, 1.04) | 0.99 (0.96, 1.02) | 1 (0.97, 1.03) | Loxenatide |  |  |
| 0.99 (0.97, 1.01) | 1.01 (0.99, 1.04) | 1 (0.98, 1.02) | 1.01 (0.99, 1.03) | 1.01 (0.99, 1.03) | Placebo |  |
| 1.01 (0.99, 1.04) | 1.04 (0.99, 1.08) | 1.02 (0.99, 1.05) | 1.03 (0.99, 1.07) | 1.03 (0.99, 1.07) | 1.02 (0.99, 1.05) | Semaglutide |

1. **Risk ratios for Diastolic Blood Pressure (mean, 95% CI)**

| Dulaglutide |  |  |  |  |  |  |
| --- | --- | --- | --- | --- | --- | --- |
| 1 (0.95, 1.06) | Exenatide |  |  |  |  |  |
| 1 (0.97, 1.03) | 1 (0.94, 1.07) | Liraglutide |  |  |  |  |
| 1.01 (0.96, 1.05) | 1.01 (0.98, 1.04) | 1.01 (0.95, 1.06) | Lixisenatide |  |  |  |
| 1 (0.95, 1.05) | 1 (0.94, 1.05) | 1 (0.94, 1.06) | 0.99 (0.95, 1.04) | Loxenatide |  |  |
| 1 (0.97, 1.04) | 1 (0.96, 1.05) | 1 (0.96, 1.05) | 1 (0.97, 1.03) | 1.01 (0.97, 1.04) | Placebo |  |
| 1.02 (0.99, 1.06) | 1.02 (0.96, 1.09) | 1.02 (0.98, 1.07) | 1.02 (0.96, 1.08) | 1.02 (0.97, 1.08) | 1.02 (0.97, 1.07) | Semaglutide |

### Table S6 Results of Scenario Analysis

| Cost/USD | | | | | |
| --- | --- | --- | --- | --- | --- |
| Year | 10 | 20 | 30 | 40 | Lifetime |
| Exenatide | 32000.39 | 37792.09 | 40894.63 | 41983.39 | 42107.35 |
| Liraglutide | 37411.34 | 43061.04 | 46305.24 | 46726.25 | 47026.46 |
| Loxenatide | 32614.5 | 38045.74 | 40612.34 | 41752.81 | 42090.63 |
| Dulaglutide | 33918.21 | 38613.04 | 42055.2 | 42539.46 | 42762.75 |
| Semaglutide | 37107.82 | 42275.99 | 45086.82 | 46388.29 | 46414.17 |
| Lixisenatide | 33335.53 | 39150.43 | 43060.85 | 43156.58 | 43388.03 |
| Utility/QALY | | | | | |
| Year | 10 | 20 | 30 | 40 | Lifetime |
| Exenatide | 7.54 | 10.94 | 12.22 | 12.55 | 12.56 |
| Liraglutide | 7.54 | 11.00 | 12.26 | 12.51 | 12.58 |
| Loxenatide | 7.55 | 11.02 | 12.33 | 12.59 | 12.65 |
| Dulaglutide | 7.55 | 10.92 | 12.22 | 12.46 | 12.5 |
| Semaglutide | 7.58 | 11.00 | 12.22 | 12.52 | 12.56 |
| Lixisenatide | 7.55 | 10.93 | 12.29 | 12.51 | 12.52 |
| NMB/USD | | | | | |
| Year | 10 | 20 | 30 | 40 | Lifetime |
| Exenatide | 63906.6 | 101428.9 | 114682 | 117793.2 | 117741.9 |
| Liraglutide | 58530.5 | 96907.4 | 109793 | 112477.3 | 113070.1 |
| Loxenatide | 63539.8 | 102175.1 | 116328.5 | 118553.4 | 118866.4 |
| Dulaglutide | 62172.95 | 100328.87 | 113523.59 | 116071.12 | 116323.7 |
| Semaglutide | 59316.14 | 97670.23 | 110478.88 | 112944.01 | 113443.68 |
| Lixisenatide | 62786.8 | 99936.3 | 113389.5 | 116045.1 | 116029 |

### Table S7 Relationship between HbA1c, Weight Control and Burden of Diabetic Comorbidities

| Parameters | Cost-saving/$ | | | Utility-gained/QALY | | |
| --- | --- | --- | --- | --- | --- | --- |
|  | mean | 95% CI | P value | mean | 95% CI | P value |
| Effective control of HbA1c | 667.32 | 89.21-1245.33 | 0.024 | 706.41 | 97.74-1315.08 | 0.023 |
| Effective control of BMI | 0.013 | 0.002-0.024 | 0.013 | 0.017 | 0.003-0.031 | 0.015 |

BMI, Body Mass Index; CI, Confidence Interval.

### Table S8 ICERs of Each Treatment in the Base-case Analysis

| ICER (USD/QALY) | VS. exenatide | VS. dulaglutide | VS. lixisenatide | VS. semaglutide | VS. liraglutide |
| --- | --- | --- | --- | --- | --- |
| loxenatide | dominant | dominant | dominant | dominant | dominant |
| exenatide | -- | -- | -- | -- | -- |
| dulaglutide | dominated | -- | -- | -- | -- |
| lixisenatide | dominated | 125300 | -- | -- | -- |
| semaglutide | 477,778 | 67611 | 54500 | -- | -- |
| liraglutide | 188,923 | 60042 | 49344 | 36000 | -- |

### eMethod 1. Details for 13 Cumulative Risk Equations

1. **Amputation**

Cumulative probability of occurrence at time t: $P_{Am}$

$$P_{Am}=\Phi(\frac{log(t)-\sum X\beta}{2.801164})$$

Where, $\Phi$ is the cumulative normal distribution function, corresponding to "NORMDIST" in Microsoft Excel;

$\sum X\beta$=20.2981−0.01600478Age−1.272875×10^−5^×(Age−45.86995)^3^+2.407403×10^−5^×(Age−61.54141)^3^−1.134528×10^−5^×(Age−79.12389)^3^−0.9533689×HbA1c+0.1235646×(HbA1c−5.6)^3^−0.1464469×(HbA1c−6.1)^3^+0.02288233×(HbA1c−8.8)^3^+0.002473774×SBP−1.480242×10^−5^×(SBP−116)3+2.846619×10^−5^×(SBP−134)^3^−1.366377×10^−5^×(SBP−153.5)^3^−0.3257868×WBC+0.005607883×(WBC−5.028605)^3^−0.009202548×(WBC−7.2)^3^+0.003594665×(WBC−10.5875)^3^+0.3328498×Hemoglobin+0.008030706×(Hemoglobin−11.38125)^3^−0.01778305×(Hemoglobin−13.64)^3^+0.009752343×(Hemoglobin−15.5)^3^+0.9007644×female−3.515543×History of amputation−1.924008×History of ulcer of skin.

Where, (x)=x if x>0, 0 otherwise; Where, [c]=1 if the subject is in group c , 0 otherwise.

WBC, White blood cell count; HbA1c, glycosylated hemoglobin; DBP, diastolic blood pressure; SBP, systolic blood pressure; T2DM, type 2 diabetes mellitus.

1. **Cataract**

Cumulative probability of occurrence at time t: $P_{Ca}$

$$P_{Ca}=\Phi(\frac{log(t)-\sum X\beta}{1.738412})$$

$\sum X\beta$=15.58701−0.09411203×Age+3.858799×10−5×(Age−45.79055)^3^−7.332382×10^−5^(Age−61.5414)^3^+3.473583×10^−5^×(Age−79.03901)^3^−0.07967085×Duration of TD2M+0.000824396×(Duration of TD2M−0.04654346)^3^−0.001662563×(Duration of TD2M−5.045859)^3^+0.0008381665×(Duration of TD2M−9.963039)^3^−0.04403888×HbA1c−0.02771952×(HbA1c−5.6)^3^+0.03285276×(HbA1c−6.1)^3^−0.005133244×(HbA1c−8.8)^3^−0.004342319×SBP+1.164939×10^−7^×(SBP−116)^3^−2.240267×10^−7^×(SBP−134)^3^+1.075328×10^−7^×(SBP−153.5)^3^+0.005686479×DBP−3.955779×10^−8^×(DBP−64.625)^3^+7.844695×10^−8^×(DBP−76.88889)^3^−3.888915×10^−8^×(DBP−89.36364)^3^−0.02348345×WBC+0.0003610212×(WBC−5.03)^3^−0.0005921174×(WBC−7.2)^3^+0.0002310962×(WBC−10.59)^3^+0.05731871×Hemoglobin+0.001758012×(Hemoglobin−11.38571)^3^−0.003898004×(Hemoglobin−13.64444)^3^+0.002139992×(Hemoglobin−15.5)^3^−0.1875367×[Ex-smoker]−0.05211328×[Non-smoker]+0.08226332×[Antihypertensive-drugs]−0.9854323×History of cataract −0.5159012×History of retinopathy.

Where, (x)=x if x>0, 0 otherwise; Where, [c]=1 if the subject is in group c , 0 otherwise.

1. **Heart failure**

Cumulative probability of occurrence at time t: $P_{HF}$

$$P_{HF}=1-[1+exp(\frac{log(t)-\sum X\beta}{1.022004})]^{-1}$$

$\sum X\beta$=9.953031−0.05613912×Age−1.04749×10^−5^×(Age−45.62902)^3^+2.003714×10^−5^×(Age−61.37714)^3^−9.562235×10^−6^×(Age−78.62834)^3^−0.08761027×Duration of TD2M+0.0006354283×(Duration of TD2M−0.04654346)^3^−0.001259727×(Duration of TD2M−4.960986)^3^+0.0006242988×(Duration of TD2M−9.963039)^3^+0.02312769×HbA1c−0.03805×(HbA1c−5.6)^3^+0.04509629×(HbA1c−6.1)^3^−0.007046296×(HbA1c−8.8)^3^+0.008674065×SBP−1.28893×10^−5^×(SBP−116)^3^+2.478712×10^−5^×(SBP−134)^3^−1.189782×10−5×(SBP−153.5)^3^+0.02084512×DBP−4.692768×10^−5^×(DBP−64.75)^3^+9.291682×10^−5^×(DBP−77)^3^−4.598913×10^−5^×(DBP−89.5)^3^+0.5125827×Triglycerides−0.1571353×(Triglycerides−0.73)^3^+0.230551×(Triglycerides−1.3)^3^−0.07341569×(Triglycerides−2.52)^3^+0.8481022×HDL−0.8385584×(HDL−0.91)^3^+1.40335×(HDL−1.27)^3^−0.5647914×(HDL−1.8045)^3^+0.01717963×BMI−0.0009786591×(BMI−20.39)^3^+0.001752005×(BMI−24.91)^3^−0.000773346×(BMI−30.63)^3^−0.2128226×WBC+0.005080483×(WBC−5.02)^3^−0.008350127×(WBC−7.2)^3^+0.003269644×(WBC−10.58736)^3^+0.2154893×Hemoglobin−0.002767901×(Hemoglobin−11.4)^3^+0.006190033×(Hemoglobin−13.66667)^3^−0.003422132×(Hemoglobin−15.5)^3^+0.1770644×female−0.2958122×status+0.01637165[Ex-smoker]+0.2246938[Non-smoker]−0.3700754×[mild CKD]−0.9045605×[mild-moderate CKD]−1.129907×[moderate severe CKD]−1.404198×[severe CKD]−1.503548×[kidney failure]−0.4162028×Insulin−0.1655605×Non insulin−0.2025641×Antihypertensive-drugs−0.5152853×History of IHD−0.3621113×History of myocardial infarction−1.143309×History of atrial fibrillation.

Where, (x)=x if x>0, 0 otherwise; Where, [c]=1 if the subject is in group c , 0 otherwise.

HDL, high-density lipoprotein; CKD, chronic kidney disease; IHD, ischemic heart disease.

1. **Ischemic heart disease**

Cumulative probability of occurrence at time t: $P_{IHD}$

$$P_{IHD}=\Phi(\frac{log(t)-\sum X\beta}{2.887255})$$

$\sum X\beta$=12.373−0.06695749×Age+3.480066×10^−5^×(Age−45.54415)^3^−6.61218×10^−5^×(Age−61.29227)^3^+3.132114×10^−5^×(Age−78.78987)^3^−0.2215785×Duration of TD2M+0.001908385×(Duration of TD2M−0.04654346)^3^−0.003783345×(Duration of TD2M−4.960986)^3^+0.00187496×(Duration of TD2M−9.963039)^3^−0.008603955×SBP+4.500772×10^−7^(SBP−116)^3^−8.729946×10^−7^×(SBP−134.1667)^3^+4.229174×10^−7^×(SBP−153.5)^3^+0.03704689×DBP−5.441537×10^−5^×(DBP−65)^3^+0.0001066541×(DBP−77)^3^−5.223875×10^−5^×(DBP−89.5)^3^+0.9058544×HDL−0.2435297×(HDL−0.915)^3^+0.406135×(HDL−1.273333)^3^−0.1626053×(HDL−1.81)^3^+0.2924694×LDL−0.05240616×(LDL−2)^3^+0.09842797×(LDL−2.96955)^3^−0.04602182×(LDL−4.0736)^3^−0.2613968×WBC+0.005757127×(WBC−5)^3^−0.009452511×(WBC−7.175)^3^+0.003695384×(WBC−10.56348)^3^+0.1612254×Hemoglobin−0.0003892057×(Hemoglobin−11.4)^3^+0.0008597028×(Hemoglobin−13.65)^3^−0.0004704971×(Hemoglobin−15.51125)^3^+0.7178127×female−0.1904222×[Ex-smoker]+0.1140562×[Non-smoker]−0.2528112×[mild CKD]−0.6643635×[mild-moderate CKD]−0.8939549×[moderate-severe CKD]−1.1436×[severe CKD]−1.222774×[kidney-failure]−0.2114733×Antihypertensive-drugs−0.6231384×Statins−0.6934588×History of heart failure+0.5835082×History of cerebrovascular disease.

Where, (x)=x if x>0, 0 otherwise; Where, [c]=1 if the subject is in group c , 0 otherwise.

LDL, Low-density lipoprotein.

1. **Myocardial infarction**

Cumulative probability of occurrence at time t: $P_{MI}$

$$P_{MI}=\Phi(\frac{log(t)-\sum X\beta}{2.230191})$$

$\sum X\beta$=12.26059−0.03579856×Age−5.736116×10^−6^×(Age−45.86995)^3^+1.084958×10^−5^×(Age−61.54141)^3^−5.11346×10^−6^×(Age−79.12115)^3^−0.03495308×Duration of TD2M−1.18837×10^−6^×(Duration of TD2M−0.04654346)^3^+2.396591×10^−6^×(Duration of TD2M−5.045859)^3^−1.208221×10^−6^×(Duration of TD2M−9.963039)^3^−0.2054818×HbA1c+0.03414138×(HbA1c−5.6)^3^−0.04046386×(HbA1c−6.1)^3^+0.006322478×(HbA1c−8.8)^3^−0.006227825×SBP−2.217406×10^−6^×(SBP−116)^3^+4.264241×10^−6^×(SBP−134)^3^−2.046836×10^−6^×(SBP−153.5)^3^+0.02343682×DBP−4.21439×10^−5^×(DBP−64.6)^3^+8.354787×10^−5^×(DBP−76.85714)^3^−4.140397×10^−5^×(DBP−89.33333)^3^+0.9748291×HDL−0.7939204×(HDL−0.91)^3^+1.328147×(HDL−1.27)^3^−0.5342268×(HDL−1.805)^3^+0.04451049×BMI−0.0001299349×(BMI−20.38)^3^+0.0002327653×(BMI−24.92)^3^−0.0001028305×(BMI−30.65667)^3^−0.2741083×WBC+0.0056222×(WBC−5.024157)^3^−0.00924382×(WBC−7.2)^3^+0.00362162×(WBC−10.57778)^3^+0.1479684×Hemoglobin+0.0009171825×(Hemoglobin−11.39235)^3^−0.00202552×(Hemoglobin−13.64)^3^+0.001108338×(Hemoglobin−15.5)^3^+0.5173726×female+0.2012889×[Ex-smoker]+0.3544502×[Non-smoker]−0.198558×[mild CKD]−0.6139906×[mild-moderate CKD]−0.9136492×[moderate-severe CKD]−1.081039×[severe CKD]−1.538418[kidney failure]−0.6108059×History of IHD−0.4667935×History of heart failure−0.5474388×History of myocardial infarction.

Where, (x)=x if x>0, 0 otherwise; Where, [c]=1 if the subject is in group c , 0 otherwise.

1. **Neuropathy**

Cumulative probability of occurrence at time t: $P_{MI}$

$$P_{PVD}=1-exp[-exp(\frac{log(t)-\sum X\beta}{1.166244})]$$

$\sum X\beta$=15.66961−0.07076835×Age+1.809499×10^−5^×(Age−45.79055)^3^−3.43075×10^−5^×(Age−61.54141)^3^+1.62125×10^−5^×(Age−79.12115)^3^−0.1320546×Duration of TD2M+0.0006558497×(Duration of TD2M−0.04654346)^3^−0.001322655×(Duration of TD2M−5.045859)^3^+0.0006668049×(Duration of TD2M−9.963039)^3^−0.1748719×HbA1c+0.004478233×(HbA1c−5.6)^3^−0.005307535×(HbA1c−6.1)^3^+0.0008293024×(HbA1c−8.8)^3^+0.6125522×HDL−0.01699118×(HDL−0.91)^3^+0.02842449×(HDL−1.27)^3^−0.01143332×(HDL−1.805)^3^+0.07861999×BMI−0.0001497577(BMI−20.37)^3^+0.0002682072×(BMI−24.91)^3^−0.0001184495×(BMI−30.65)^3^−0.2534007×WBC+0.005037967×(WBC−5.026583)^3^−0.008258439×(WBC−7.2)^3^+0.003220471×(WBC−10.6)^3^+0.2629841×Hemoglobin−0.000898584×(Hemoglobin−11.4)^3^+0.001991456×(Hemoglobin−13.65)^3^−0.001092872×(Hemoglobin−15.5)^3^+0.7126738×female+0.1445479×[Ex-smoker]+0.8039165×[Non-smoker]−0.1557765×[mild CKD]−0.873991×[mild-moderate CKD]−0.9208683×[moderate-severe CKD]−1.351951×[severe CKD]−2.037225×[kidney failure]−2.611367×History of amputation−1.374109×History of ulcer of skin.

Where, (x)=x if x>0, 0 otherwise; Where, [c]=1 if the subject is in group c , 0 otherwise.

1. **Peripheral vascular disease**

Cumulative probability of occurrence at time t: $P_{PVD}$

$$P_{MI}=\Phi(\frac{log(t)-\sum X\beta}{3.257492})$$

$\sum X\beta$=14.33607−0.1805239×Duration of TD2M+0.001101617×(Duration of TD2M−0.04654346)^3^−0.00218394×(Duration of TD2M−4.960986)^3^+0.001082323×(Duration of TD2M−9.963039)^3^−0.3248836×HbA1c+0.008286954×(HbA1c−5.6)^3^−0.009821576×(HbA1c−6.1)^3^+0.001534621×(HbA1c−8.8)^3^+0.001098733×SBP−1.169469×10^−5^×(SBP−116)^3^+2.242102×10^−5^×(SBP−134)^3^−1.072634×10^−5^×(SBP−153.625)^3^+0.1214197×BMI−0.000815454×(BMI−20.36)^3^+0.00146043×(BMI−24.9)^3^−0.0006449759×(BMI−30.64)^3^−0.3202284×WBC+0.0108545×(WBC−5.03)^3^−0.01778222×(WBC−7.2)^3^+0.006927725×(WBC−10.6)^3^+0.1917622×Hemoglobin+0.003465902×(Hemoglobin−11.375)^3^−0.007659024×(Hemoglobin−13.63333)^3^+0.004193122×(Hemoglobin−15.5)^3^+0.9294109×female−1.109639×status−0.01937676×[mild-CKD]−0.6279954×[mild-moderate CKD]−0.845451×[moderate-severe CKD]−1.812507×[severe CKD]−1.992212×[kidney failure].

Where, (x)=x if x>0, 0 otherwise; [c]=1 if the subject is in group c , 0 otherwise; status, T2DM=1, 0 otherwise.

1. **Renal failure**

Cumulative probability of occurrence at time t: $P_{RF}$

$$P_{RF}=1-[1+exp(\frac{log(t)-\sum X\beta}{0.7981171})]^{-1}$$

$\sum X\beta$=6.166196−0.004849129×Age−1.901472×10^−5^×(Age−45.86995)^3^+3.604495×10^−5^×(Age−61.54141)^3^−1.703024×10^−5^×(Age−79.03901)^3^−0.1409224×Duration of TD2M+0.0007897111×(Duration of TD2M−0.04654346)^3^−0.00156559×(Duration of TD2M−4.960986)^3^+0.0007758793×(Duration of TD2M−9.963039)^3^−0.02053097×HbA1c−0.02178857×(HbA1c−5.6)^3^+0.02582349×(HbA1c−6.1)^3^−0.00403492×(HbA1c−8.8)^3^−0.01317253×SBP−5.375805×10^−6^×(SBP−116)^3^+1.033809×10^−5^(SBP−134)^3^−4.962282×10^−6^×(SBP−153.5)^3^+0.02357291×DBP−3.271212×10^−5^×(DBP−64.66667)^3^+6.498808×10^−5^×(DBP−77)^3^−3.227596×10^−5^×(DBP−89.5)^3^−0.4526705×Triglycerides+0.08718189×(Triglycerides−0.725)^3^−0.1285051×(Triglycerides−1.299)^3^+0.04132321×(Triglycerides−2.51)^3^−0.008416627×LDL−0.03346604×(LDL−1.96452)^3^+0.06281429×(LDL−2.9418)^3^−0.02934825×(LDL−4.0562)^3^−0.154757×WBC+0.004885142×(WBC−5.03)^3^−0.008003012×(WBC−7.2)^3^+0.00311787×(WBC−10.6)^3^+0.5700462×Hemoglobin−0.00978487×(Hemoglobin−11.4)^3^+0.02168539×(Hemoglobin−13.65)^3^−0.01190052×(Hemoglobin−15.5)^3^+1.081168×female−0.2573695×status−0.1490706×[Ex-smoker]−0.05581874×[Non-smoker]−0.2720151×Antihypertensive-drugs−0.1631902History of cataract−0.6716275History of heart failure−0.4899921History of retinopathy−0.3150657History of PVD−0.1800056History of cerebrovascular disease.

Where, (x)=x if x>0, 0 otherwise; [c]=1 if the subject is in group c , 0 otherwise; status, T2DM=1, 0 otherwise.

1. **Skin ulcer**

Cumulative probability of occurrence at time t: $P_{Re}$

$$P_{Re}=\Phi(\frac{log(t)-\sum X\beta}{2.202554})$$

$\sum X\beta$=12.4049−0.02984757×Age−1.813997×10^−5^(Age−45.86995)^3^+3.430832×10^−5^×(Age−61.54141)^3^−1.616835×10^−5^×(Age−79.12389)^3^−0.1465888×Duration of TD2M+0.001770892×(Duration of TD2M−0.04654346)^3^−0.003571364×(Duration of TD2M−5.045859)^3^+0.001800472×(Duration of TD2M−9.963039)^3^+0.0008940611×HbA1c−0.048112×(HbA1c−5.6)^3^+0.05702163×(HbA1c−6.1)^3^−0.008909629×(HbA1c−8.8)^3^−0.006295492×DBP−1.635404×10^−5^×(DBP−64.6)^3^+3.242095×10^−5^×(DBP−76.85714)^3^−1.606691×10^−5^×(DBP−89.33333)^3^+0.0648033×BMI−0.0006703127×(BMI−20.38)^3^+0.001201169×(BMI−24.92)^3^−0.0005308558×(BMI−30.65267)^3^−0.2250905×WBC+0.004247334×(WBC−5.026667)^3^−0.006978362×(WBC−7.2)^3^+0.002731028×(WBC−10.58)^3^+0.2948477×Hemoglobin+0.0003555979×(Hemoglobin−11.3877)^3^−0.0007869016×(Hemoglobin−13.64167)^3^+0.0004313036×(Hemoglobin−15.5)^3^+0.4907979×female−0.5539779×status−0.324728×Non-insulin−1.875538×History of amputation−0.3460361×History of cerebrovascular disease−2.192475×History of ulcer of skin.

Where, (x)=x if x>0, 0 otherwise; [c]=1 if the subject is in group c , 0 otherwise; status, T2DM=1, 0 otherwise.

1. **Retinopathy**

Cumulative probability of occurrence at time t: $P_{Re}$

$$P_{Re}=\Phi(\frac{log(t)-\sum X\beta}{2.643858})$$

$\sum X\beta$=17.63971−0.8386386×HbA1c+0.1153007×(HbA1c−5.6)^3^−0.1366527×(HbA1c−6.1)^3^+0.02135198×(HbA1c−8.8)^3^−0.01942327×SBP+4.726123×10^−7^×(SBP−116)^3^−9.06644×10^−7^×(SBP−134)^3^+4.340317×10^−7^×(SBP−153.6)^3^−0.564101×HDL+0.8751246×(HDL−0.91)^3^−1.467036×(HDL−1.27)^3^+0.5919114×(HDL−1.80225)^3^+0.06123235×BMI−0.0001372454×(BMI−20.37)^3^+0.0002457984×(BMI−24.91)^3^−0.000108553×(BMI−30.65)^3^+0.2032228×Hemoglobin+0.002140977×(Hemoglobin−11.38333)^3^−0.004764156×(Hemoglobin−13.65)^3^+0.002623179×(Hemoglobin−15.5)^3^+0.5429286×female−0.6915248×status−0.04408495×[mild CKD]−0.3462679×[mild-moderate CKD]−0.6017722×[moderate-severe CKD]−1.052448×[severe CKD]−1.806614×[kidney failure]−0.369624×Non insulin+0.4482381×Antihypertensive-drugs−0.2948715×History of cataract+0.4902719×History of IHD.

Where, (x)=x if x>0, 0 otherwise; [c]=1 if the subject is in group c , 0 otherwise.

1. **Stroke**

Cumulative probability of occurrence at time t: $P_{Stroke}$

$$P_{Stroke}=\Phi(\frac{log(t)-\sum X\beta}{2.37858})$$

$\sum X\beta$=15.82026−0.0704093×Age+1.485588×10^−5^×(Age−45.78782)^3^−2.823105×10^−5^×(Age−61.54141)^3^+1.337517×10^−5^×(Age−79.03901)^3^−0.1366587×Duration of TD2M+0.001241157×(Duration of TD2M−0.04654346)^3^−0.002503046×(Duration of TD2M−5.045859)^3^+0.001261889×(Duration of TD2M−9.963039)^3^+0.08792962×HbA1c−0.03425862×(HbA1c−5.6)^3^+0.04060281×(HbA1c−6.1)^3^−0.006344189×(HbA1c−8.8)^3^−0.01030808×SBP−4.042218×10^−6^×(SBP−116)^3^+7.805663×10^−6^×(SBP−134)^3^−3.763445×10^−6^×(SBP−153.3333)^3^+0.007178522×DBP−3.620796×10^−5^×(DBP−64.6)^3^+7.164348×10^−5^×(DBP−76.83333)^3^−3.543552×10^−5^×(DBP−89.33333)^3^+0.6314516×HDL−0.3696312×(HDL−0.91)^3^+0.6183549×(HDL−1.27)^3^−0.2487238×(HDL−1.805)^3^+0.04083765×BMI−0.0002714759×(BMI−20.39)^3^+0.0004861972×(BMI−24.93)^3^−0.0002147213×(BMI−30.67)^3^−0.2198664×WBC+0.004713937×(WBC−5.015)^3^−0.007767682×(WBC−7.1975)^3^+0.003053745×(WBC−10.56653)^3^+0.04818864×Hemoglobin+0.003859686×(Hemoglobin−11.4)^3^−0.008553899×(Hemoglobin−13.65)^3^+0.004694213×(Hemoglobin−15.5)^3^+0.3607541×female−0.3148034×status−1.31932×History of cerebrovascular disease−0.7094421×History of atrial fibrillation.

Where, (x)=x if x>0, 0 otherwise; [c]=1 if the subject is in group c , 0 otherwise; status, T2DM=1, 0 otherwise

1. **Diabetes**

Cumulative probability of occurrence at time t: $P_{DM}$

$$P_{DM}=\Phi(\frac{log(t)-\sum X\beta}{1.604527})$$

$\sum X\beta$=4.911377+0.6414919×HbA1c−5.706087×(HbA1c−5.5)^3^+13.3142×(HbA1c−5.9)^3^−7.608117×(HbA1c−6.2)^3^−0.006145417×SBP−1.1125×10^−6^×(SBP−116)^3^+2.183909×10^−6^×(SBP−133.6)^3^−1.071409×10^−6^×(SBP−151.875)^3^−0.3932508×Triglycerides+0.1237671×(Triglycerides−0.7)^3^−0.18115×(Triglycerides−1.21)^3^+0.05738291×(Triglycerides−2.31)^3^+0.2087609×HDL−0.06976457×(HDL−0.95)^3^+0.1162743×(HDL−1.33)^3^−0.04650972×(HDL−1.9)^3^+0.08693763×LDL−0.00724271×(LDL−1.956849)^3^+0.01369647×(LDL−2.95)^3^−0.00645376×(LDL−4.06456)^3^+0.008889837×BMI−0.0003679625×(BMI−20.18)^3^+0.000669188×(BMI−24.725)^3^−0.0003012255×(BMI−30.27695)^3^−0.08623678×WBC+0.000575842×(WBC−4.85)^3^−0.0009413155×(WBC−6.885)^3^+0.0003654735×(WBC−10.09136)^3^+0.189371×Hemoglobin−0.01186573×(Hemoglobin−11.4)^3^+0.02570909×(Hemoglobin−13.5)^3^−0.01384335×(Hemoglobin−15.3)^3^−0.07984426×[Ex-smoker]+0.09417903×[Non-smoker]+0.2138855×Statins−0.2382902×History of heart failure−0.2052062×History of myocardial infarction−0.5095882×History of PVD.

Where, (x)=x if x>0, 0 otherwise; [c]=1 if subject is in group c , 0 otherwise; status, T2DM=1, 0 otherwise.

1. **Death**

Cumulative probability of occurrence at time t: $P_{DM}$

$$P_{RF}=1-[1+exp(\frac{log(t)-\sum X\beta}{0.5818242})]^{-1}$$

$\sum X\beta$=5.654104−0.04733169×Age+2.087535×10^−6^×(Age−45.86995)^3^−3.948179×10^−6^×(Age−61.54141)^3^+1.860644×10^−6^×(Age−79.12389)^3^−0.02389083×Duration of TD2M+0.000120329×(Duration of TD2M−0.04654346)^3^−0.0002426679×(Duration of TD2M−5.045859)^3^+0.0001223389×(Duration of TD2M−9.963039)^3^+0.03401475×HbA1c−0.02138119×(HbA1c−5.6)^3^+0.02534067×(HbA1c−6.1)^3^−0.003959479×(HbA1c−8.8)^3^+0.008013911×SBP−4.678043×10^−6^×(SBP−116)^3^+8.996237×10^−6^×(SBP−134)^3^−4.318194×10^−6^×(SBP−153.5)^3^−0.006942038×DBP−5.914667×10^−6^×(DBP−64.6)^3^+1.172549×10^−5^×(DBP−76.85714)^3^−5.810822×10^−6^×(DBP−89.33333)^3^+0.2701938×HDL−0.2718915×(HDL−0.91)^3^+0.4550176×(HDL−1.27)^3^−0.1831262×(HDL−1.8045)^3^+0.0579741×LDL−0.02429757×(LDL−1.9645)^3^+0.04555841×(LDL−2.94)^3^−0.02126083×(LDL−4.054833)^3^+0.09031361×BMI−0.0007535335×(BMI−20.38)^3^+0.001350574×(BMI−24.92)^3^−0.0005970405×(BMI−30.65)^3^−0.06176844×WBC−5.717377×10^−5^×(WBC−5.03)^3^+9.377172×10^−5^×(WBC−7.2)^3^−3.659796×10^−5^×(WBC−10.59)^3^+0.2638521×Hemoglobin−0.006302378×(Hemoglobin−11.37778)^3^+0.01391775×(Hemoglobin−13.63333)^3^−0.007615374×(Hemoglobin−15.5)^3^+0.5005344×female−0.0847309×status+0.1975923×[Ex-smoker]+0.1776321×[Non-smoker]+0.140743×[mild CKD]+0.02327084×[mild-moderate CKD]−0.07588053×[moderate-severe CKD]−0.2925816×[severe CKD]−0.3862744×[kidney failure]−0.2425206×Non insulin−0.07347614×Antihypertensive-drugs+0.05791233×Statins−0.08061887×History of IHD−0.3327469×History of heart failure−0.1691838×History of PVD−0.1463592×History of cerebrovascular disease−0.2695514×History of atrial fibrillation.

Where, (x)=x if x>0, 0 otherwise; [c]=1 if the subject is in group c , 0 otherwise; status, T2DM=1, 0 otherwise

### eMethod 2. Model Validation

The RECODe model predicts risks at a specified period of 10 years; for comparison, CHIME, UKPDS-OM2, and RECODe models were assessed against CMS participants enrolled

from 2006 to 2008 and followed until December 31, 2017. CHIME and UKPDS-OM2 models against the CHARLS validation cohort at 6 years of follow-up (wave 4 conducted from July to September 2018). Since UKPDS-OM2 and RECODe do not predict for participants with prediabetes, for our main analyses, we compared all models against CMS and CHARLS participants with type 2 diabetes only (heart rate data were unavailable for CHARLS participants). Model discrimination was assessed using the C-statistic at 10 years for CMS and 6 years for CHARLS with confidence intervals estimated from 100 bootstrap replications. We assessed calibration through the slope and intercept of the line between predicted and observed probabilities of each outcome by deciles of risk, with fewer centiles than deciles used if fewer than five events were observed per group to prevent unstable inferences. We also measured the goodness of fit between predicted and observed endpoints using the root mean square percentage error (RMSPE), where lower scores indicate better fit, and present scatterplots of predicted versus observed endpoints along with the coefficient of determination (R^2^). Since the CHARLS data only reported mortality, ischemic heart disease, cerebrovascular disease, renal failure, cataracts, and diabetes status, we also performed validation against published trial data, in keeping with the performance assessment strategy used in most diabetes outcomes models that lacked an individual-level validation cohort. We used the published baseline characteristics of the trial participants to generate a simulated cohort for the duration of each respective trial, with separate cohorts for each arm of the trial to model differing treatment effects between intervention and control arms.

Internal and external validation statistics for CHIME, UKPDS-OM2, and RECODe for diabetes.


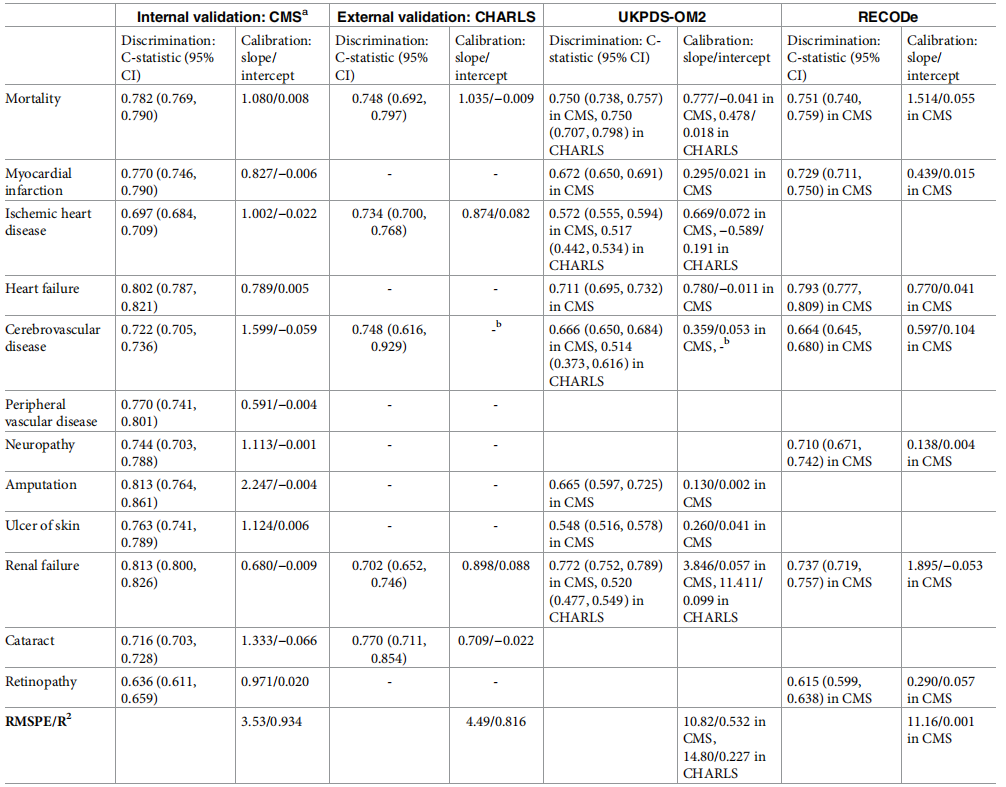


a CMS participants enrolled from 2006–2008.

b Only 3 centiles for calibration.

CHARLS, China Health and Retirement Longitudinal Study; CHIME, Chinese Hong Kong Integrated Modeling and Evaluation; CI, confidence interval; CMS, Clinical Management System; RECODe, Risk Equations for Complications Of type 2 Diabetes; RMSPE, root mean square percentage error; UKPDS-OM2, UK Prospective Diabetes Study Outcomes Model 2.

Calibration plots of CHIME, UKPDS-OM2, and RECODe model for diabetes


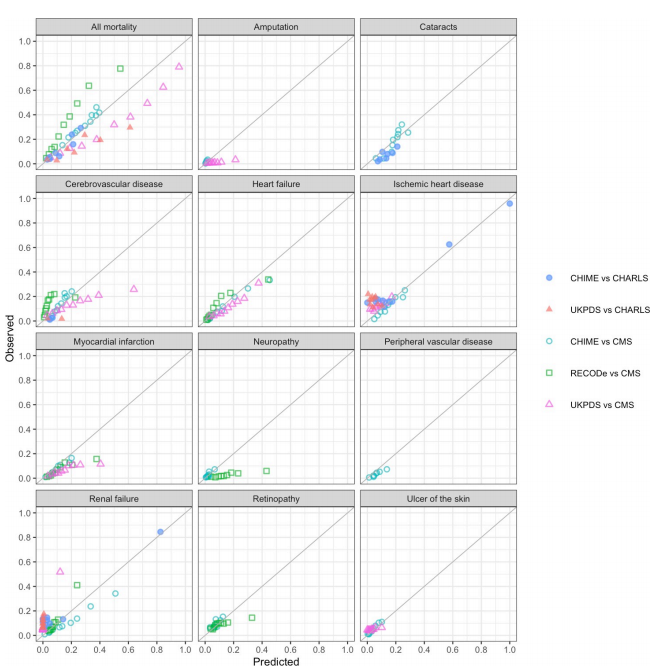


Predictions using CHIME, UKPDS-OM2, and RECODe are presented if available. Points are displayed for deciles of predicted and observed event rates, with fewer centiles than deciles used if fewer than 5 events were observed per group to prevent unstable inferences. CHARLS, China Health and Retirement Longitudinal Study; CHIME, Chinese Hong Kong Integrated Modeling and Evaluation; CMS, Clinical Management System; RECODe, Risk Equations for Complications Of type 2 Diabetes; UKPDS-OM2, United Kingdom Prospective Diabetes Study Outcomes Model 2.

Validation of CHIME and UKPDS-OM2 models against CMS cohort.


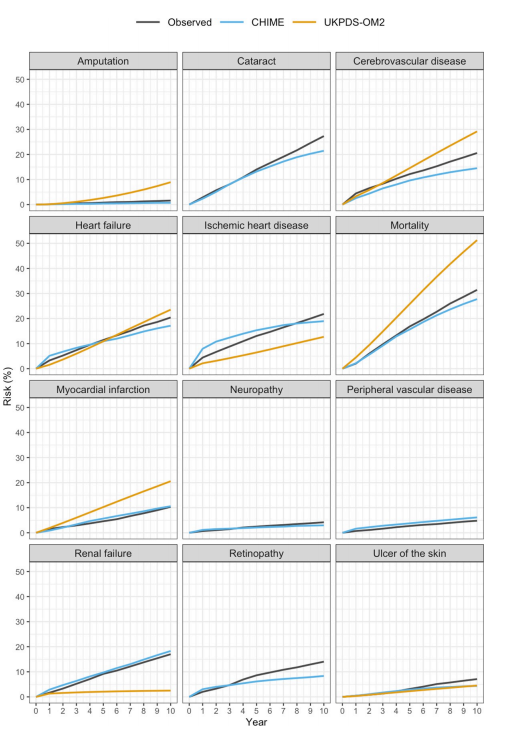


Predicted event rates from UKPDS and CHIME models against the observed event rates over time for CMS participants with diabetes. CHIME, Chinese Hong Kong Integrated Modeling and Evaluation; CMS, Clinical Management System; UKPDS-OM2, UK Prospective Diabetes Study Outcomes Model 2.

Validation of CHIME, UKPDS-OM2, and RECODE models against simulated cohorts from trials.


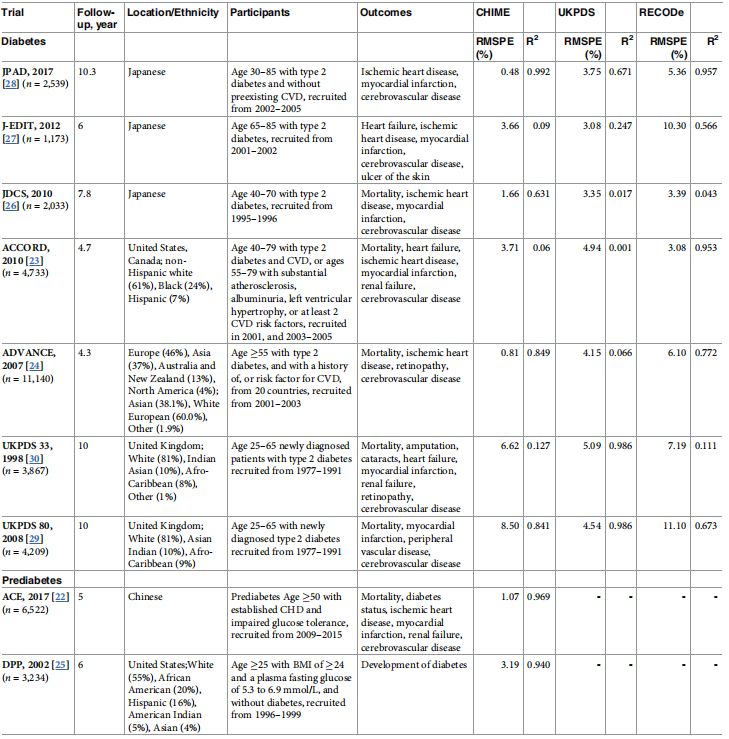


*Simulated 100,000 participants for validation.

RMSPE, root mean square percentage error; CHD, coronary heart disease; CVD, cardiovascular disease; BMI, body mass index; eGFR, estimated glomerular filtration rate; ACE, Acarbose Cardiovascular Evaluation [22]; ACCORD, Action to Control Cardiovascular Risk in Diabetes [23]; ADVANCE, Action in Diabetes and Vascular disease: preterAx and diamicroN-MR Controlled Evaluation [24]; DPP, Diabetes Prevention Program [25]; JDCS, Japan Diabetes Complications Study [26]; J-EDIT, Japan Elderly Diabetes Intervention Trial [27]; JPAD, Japanese Primary Prevention of Atherosclerosis with Aspirin for Diabetes trial [28]; UKPDS, United Kingdom Prospective Diabetes Study [29,30].

External validation of CHIME, UKPDS-OM2, and RECODe against trial data.


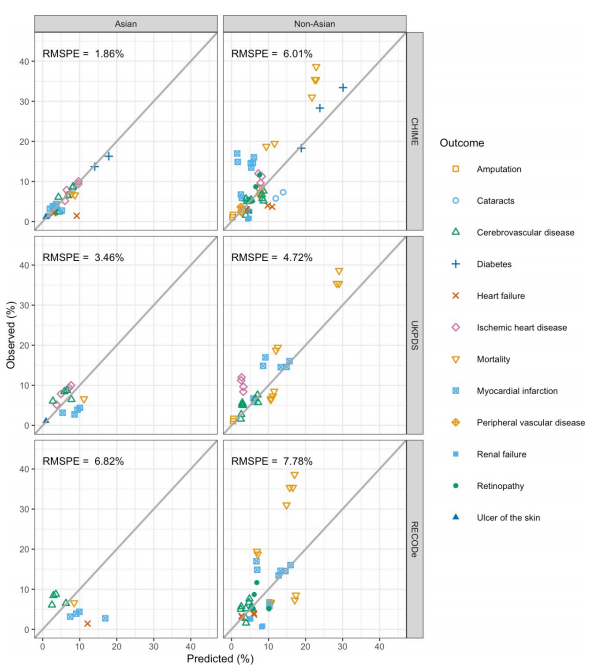


Predicted percentage of events from CHIME, UKPDS-OM2, and RECODe against observed percentage in Asian trials (ACE, JPAD, J-EDIT, and JDCS) and non-Asian trials (ACCORD, ADVANCE, DPP, UKPDS 33, and UKPDS 80).

ACCORD, Action to Control Cardiovascular Risk in Diabetes; ACE, Acarbose Cardiovascular Evaluation; ADVANCE, Action in Diabetes and Vascular disease: preterAx and diamicroN-MR Controlled

Evaluation; CHIME, Chinese Hong Kong Integrated Modeling and Evaluation; DPP, Diabetes Prevention Program; JDCS, Japan Diabetes Complications Study; J-EDIT, Japan Elderly Diabetes Intervention Trial; JPAD, Japanese Primary Prevention of Atherosclerosis with Aspirin for Diabetes; RECODe, Risk Equations for Complications Of type 2 Diabetes; RMSPE, root mean square percentage error; UKPDS-OM2, UK Prospective Diabetes Study Outcomes Model 2; UKPDS 33, UK Prospective Diabetes Study 33; UKPDS 80, UK Prospective Diabetes Study 80

**eMethod 3. NMA Process**

Random-effect Bayesian models estimated risk ratios (RRs) of the changing rates between the six treatments via Markov chain Monte Carlo algorithms. The reference for glycated hemoglobin and BMI was set as lixisenatide in GetGoal-X, which was the trial with the largest sample size in our network, and semaglutide in SUSTAIN-7 was used as the reference for SBP and DBP for the same reason. Noninformative priors were used to allow the observed trial data to explain effect estimates ^[1]^. We used the gemtc package ^[2]^ in R, version 4.1.0 ^[3]^ with 4 parallel Markov chains consisting of 50,000 samples after a 10,000 sample burn-in. The convergence of Markov chains was checked by trace plots and Gelman-Rubin diagnostic statistics. The design-by-treatment approach was used to check the consistency in the entire NMA, and the global inconsistency of the study was assessed with the I^2^ (chi-square test) statistic. The local inconsistency was evaluated by node-splitting analysis, which was used to assess the inconsistency of the model by separating evidence on a particular comparison into direct and indirect evidence. The significance level was α=0.05 for statistical tests.

**References**

[1] Dias S, Ades A E, Welton N J, et al. Network meta-analysis for decision-making[M]. John Wiley & Sons, 2018.

[2] Shim S R, Kim S, Lee J, et al. Network meta-analysis: application and practice using R software[J]. Epidemiology and health, 2019,41.

[3] Computing R. R: A language and environment for statistical computing[J]. Vienna: R Core Team, 2013.
